# Supplementary material for: Determining counselling communication strategies associated with successful quits in the National Health Service community pharmacy Stop Smoking programme in East London: a focused ethnography using recorded consultations
Source: BMJ Open. 2017 Oct 27;7(10):e015664. doi: 10.1136/bmjopen-2016-015664 (PMC5665230; doi:10.1136/bmjopen-2016-015664)
Supplement: Author's manuscript [file bmjopen-2016-015664.draft_revisions.pdf]

BMJ Open

BMJ Open is committed to open peer review. As part of this commitment we make the peer review history of every article we publish publicly available.

When an article is published we post the peer reviewers' comments and the authors' responses online. We also post the versions of the paper that were used during peer review. These are the versions that the peer review comments apply to.

The versions of the paper that follow are the versions that were submitted during the peer review process. They are not the versions of record or the final published versions. They should not be cited or distributed as the published version of this manuscript.

BMJ Open is an open access journal and the full, final, typeset and author-corrected version of record of the manuscript is available on our site with no access controls, subscription charges or pay-per-view fees (http://bmjopen.bmj.com).

If you have any questions on BMJ Open's open peer review process please email editorial.bmjopen@bmj.com

BMJ Open

Defining effective communication strategies in the NHS community pharmacy stop smoking programme: an ethnographic study of consultations with smokers

Journal:	*BMJ Open*
Manuscript ID	bmjopen-2016-015664
Article Type:	Research
Date Submitted by the Author:	20-Dec-2016
Complete List of Authors:	Rivas, Carol; University of Southampton, Faculty of Health Sciences; Queen Marys School of Medicine and Dentistry, Centre for Primary Care and Public Health<br>Sohanpal, Ratna; Queen Marys School of Medicine and Dentistry, Centre for Primary Care and Public Health<br>MacNeill, Virginia; University of Oxford<br>Steed, Liz; Queen Marys School of Medicine and Dentistry, Centre for Primary Care and Public Health<br>Edwards, Elizabeth; Queen Mary University of London, Centre for Primary Care and Public Health<br>Antao, Laurence; Queen Mary University of London<br>Griffiths, Chris; Queen Mary University of London<br>Eldridge, Sandra; Queen Mary University of London, Centre for Primary Care and Public Health<br>Taylor, Stephanie; Queen Mary University of London, Primary Care and Public Health<br>Walton, Robert; Queen Mary University of London
**Primary Subject Heading**:	Smoking and tobacco
Secondary Subject Heading:	Health services research, Public health, Qualitative research, Patient-centred medicine
Keywords:	Smoking cessation, Community Pharmacies, QUALITATIVE RESEARCH, Ethnography, Communication

SCHOLARONE™
Manuscripts

Defining effective communication strategies in the NHS community pharmacy stop smoking programme: an ethnographic study of consultations with smokers

Carol Rivas[12]

Ratna Sohanpal[2]

Virginia MacNeill[3]

Liz Steed[2]

Elizabeth Edwards[2]

Laurence Antao[2]

Chris Griffiths[2]

Sandra Eldridge[2]

Stephanie Taylor[2]

Robert Walton[2]

[1]Faculty of Health Sciences, University of Southampton, UK

[2]Centre for Primary Care and Public Health, , Queen Mary University of London, London, UK

[3]University of Oxford, UK

Corresponding author:

Carol Rivas

Senior Research Fellow

University of Southampton

School of Health Sciences

Highfield, Building 67

Southampton, SO17 1BJ

Tel: 023 8059 8532

Email: c.a.rivas@soton.ac.uk

c.a.rivas@qmul.ac.uk

For peer review only - http://bmjopen.bmj.com/site/about/guidelines.xhtml

For peer review only

ABSTRACT (300/300words)

Objectives: To determine communication strategies associated with smoker retention and smoking cessation in the National Health Service community pharmacy Stop Smoking programme to inform best practice.

Setting: Eleven community pharmacies in three inner east London boroughs.

Participants:

Fifty-three smokers provided 158 consultation recordings with 11 advisers. We selected 16 pairs of smokers who either quit at four weeks or did not quit, matched on gender, ethnicity, age and smoking intensity. Each pair member provided 1-3 recordings over 5-6 weeks (n=47, 9 advisers).

Method: Qualitative thematic content analysis of consultation transcripts, augmented by quantitative content analysis.

Results: The quantitative data revealed an adviser core strategy set leaning to the voice of medicine, omitting explicit motivational interviewing. This was balanced in eventual quitters only by additional core strategies involving support, 'open door' invitations and willpower talk. Qualitative differences between quitter and non-quitter consultations were also evident in the three overarching themes arising from thematic analysis. A subtheme of 'Negotiating the smoker-adviser relationship' was lifeworld talk, facilitated by social familiarity between smokers and advisers, and apparently used by advisers to judge smoker quit likelihood. 2) The 'roles of the adviser and the smoker in the quit attempt' them indicated advisers well-meaningly modified core strategies to suit their prejudgements. This could lead to misalignment in non-quitters through inappropriately overemphasised praise and managing expectations talk and underplayed talk on willpower and adviser support, and inconsistencies in implementation of NCSCT training recommendations. 3) 'Smoker and adviser misalignment on reasons for smoking, relapsing and quitting', was the third theme.

Discussion: Some core strategies and adviser prejudgements of likely smoker success were premised on a 'voice of medicine' power differential. Advisers should maximise the community setting benefits and their social familiarity with smokers to restore the balance to the 'voice of the lifeworld' and enable more person-centred care.

Keywords: Smoking cessation, Community Pharmacies, Qualitative research, Ethnography, Communication

For peer review only - http://bmjopen.bmj.com/site/about/guidelines.xhtml

Strengths and limitations of this study

- To our knowledge this is the first study to consider verbal interactions in real world consultations between community pharmacy advisers and smokers in the NHS community pharmacy Stop Smoking programme
- 32 smokers from a larger sample were matched on demographic variables to provide 16 matched pairs of four-week quitters and non-quitters, enhancing confidence in findings. Theme saturation with rich data collection was achieved.
- The findings may not be transferable to other pharmacies, particularly in less disadvantaged areas, or to over the counter community pharmacy interactions since the consultations took place in dedicated consultation rooms.
- Our study findings are limited to interactions during the consultation, as appropriate to the methods chosen, and we did not systematically explore previous quit attempts and their influence on results.

Introduction

UK community pharmacies have adopted public health and healthy living tasks that were previously the domain of the general practitioner.[1] These 'enhanced services' are embedded in UK health service policy and embraced by pharmaceutical professional bodies.[2-9] Community pharmacies have great potential to provide such health care, particularly for people from disadvantaged groups,[10] given their community setting, long opening hours, accessibility, familiarity and informality.[11-13] Pharmacy enhanced services therefore provide an opportunity to address health inequalities and may free general practice time for patients with more complex problems.[14]

The NHS Stop Smoking programme was the most frequently delivered enhanced community pharmacy service in England in 2012[15] and its effectiveness measured by smoker self-reported 4-week quit rates was comparable to that in general practice (49% vs. 47%).[15,16] But neither general practice nor community pharmacies achieve the suggested optimal quit rate of 70%,[7] and there is considerable variation in rates within the same setting.[16] Moreover, the proportion of smokers enrolling on the Stop Smoking programme in community pharmacies rather than GP surgeries and other settings has remained stable (between 18-21%) since the programme began in 2006 despite government efforts to shift some of the GP workload to them.[7,16] Our study aims to improve both service uptake and quit rates in the community pharmacy Stop Smoking programme.

Enhanced services have necessitated a change in mind set for pharmacy staff with a move towards the person-centred care model currently advocated within health services.[17-24] Pharmacy staff tend to be inadequately trained in this model,[22] and may lack necessary consultation skills.[22,24] We therefore used an ethnographic approach, using recordings of naturalistic (real world) consultations to explore verbal interactions between pharmacy Stop Smoking advisers and smokers that might affect smoker engagement, maintenance within the pharmacy programme and success in quitting.

Methods

Pharmacies and advisers

Consultations were recorded between November 2013 and May 2014 at community pharmacies contracted by Public Health England to provide the Stop Smoking programme in three inner East London boroughs (Newham, Tower Hamlets, the City and Hackney). These areas are economically disadvantaged compared with other areas of London and with England as a whole[25] and have relatively poor NHS Stop Smoking programme attendance.[13,15,16,] We only included community

For peer review only - http://bmjopen.bmj.com/site/about/guidelines.xhtml

pharmacy chains or independent single pharmacies, which are distinct from those in institutional settings such as NHS hospitals. Consultations had to take place in a private room.

We used maximum variation sampling by borough, pharmacy size and length of time on the Stop Smoking programme. Within pharmacies, we invited any staff members certificated through the National Centre for Smoking Cessation and Training (NCSCT) programme, explained the study, took consent, and trained them in research methods and governance, so that they could take smoker service user consent and record, securely store and transfer their own consultations to the research team. This meant the natural rhythm of the service was not disrupted. Ethical approval for the study was obtained from the NRES Committee South Central, Berkshire B (reference number 13/SC/0189).

Smokers

All adult smokers recruited onto the NHS Stop Smoking programme during the course of the study were eligible. Smokers were designated as having quit if they self-reported a smoke-free status at 4 weeks after their set quit date and provided an adviser with an expired carbon monoxide (CO) reading of less than 7 parts per million using a CO monitor. Smokers lost to follow up at four weeks were considered still smoking following the Russell criteria.[26]

Data collection

Community pharmacy NHS Stop Smoking programme consultations and prescriptions for medication may continue for up to 12 weeks with formal consultations weekly or two-weekly and informal drop-ins between these times as needed.[7,27] The core period for the programme ends at five to six weeks which tends to equate with the fourth week after the quit date, usually set at the first consultation.[7,27]

Stop Smoking advisers consented eligible smokers and audiorecorded:

1. their first consultation, which tends to set the parameters for the remaining sessions, includes the smoker setting a proximal quit date, and is usually up to three times as long as subsequent sessions (averaging 15 minutes vs 5 minutes);[28]
2. the consultation at 2 weeks post proposed quit date where the adviser explores and attempts to solve any issues in the quit attempt;
3. the consultation four weeks post proposed quit date at which the smoker's quit status is formally designated (as the 4-week quit status) for Public Health England audit statistics and pharmacy remuneration.[29]

We call these weeks 1, 2 and 4 for simplicity. Recordings were anonymised and transcribed as they were received. Pseudo-anonymised metadata (such as demographic data and smoking intensity for the smokers, and training and demographic data for the advisers) were entered with anonymised recordings and transcripts onto a secure clinical trials database.

Consultations analysis

We refer to quitters and non-quitters throughout this paper but in consultations 1 and 2 this is a retrospective designation for *eventual* quitters and non-quitters. We matched 16 pairs of 4-week quitters and non-quitters (i.e. 32 smokers) on gender, ethnicity, age (according to UK Office for National Statistics [ONS] age bands), and smoking intensity (fewer than 10 cigarettes a day, 10-20 a day, more than 20 a day). We had initially aimed for 20 matched pairs but obtained saturation at this lower number. We considered the impact of adviser experience, role in the pharmacy, and smoker retention rates on quit success.

For peer review only - http://bmjopen.bmj.com/site/about/guidelines.xhtml
For peer review only

Following a qualitative content thematic analysis approach as outlined in Clark and Braun[30] and Rivas[31], the core team immersed themselves in the data, reading and re-reading each consultation transcription and meeting to discuss these. CR led inductive development of themes from the data, constantly comparing emerging themes with each other and also comparing data within themes to ensure each one was discrete and representative of the relevant data. CR operationalised the themes, for a second researcher (RS) to code 20% of the data independently. Differences were reconciled by discussion, until an acceptable Cohen's kappa statistic of 76% was obtained. Some themes reflect the intention to also analyse the micro-detail of the consultation talk using conversation analysis, to be reported in subsequent papers.

For a broad quantitative overview of the data, we compared the frequency of themes between and within successful and unsuccessful quitter consultations. We counted the number of times a theme was coded overall and divided this by the number of consultations in which it occurred to get an idea of the intensity within a consultation. We also considered the number of smokers for whom each theme was cited, to get an idea of which themes formed a core set for advisers. This process enabled us to focus on themes that were of high or moderate intensity and core usage and associated with particular outcomes, as well as strategies rarely used but that might be important for service improvement. Given the small numbers involved, tests of significance are not reported here.

Findings

Participants

Thirty-nine percent (11/28) of recruited advisers provided 158 audiorecordings from 53 smokers. Considering only the 16 matched pairs, these were seen by 9 of the 11 advisers, with a median of three smokers each (range 1-7). Table 1 compares baseline characteristics of the matched pairs to the total sample and Table 2 provides further detail on the matched pairs. Whilst women were under-represented in the matched pairs, other characteristics related to cessation outcome were broadly similar. This includes medication use; though we did not monitor for this systematically, the transcripts indicate that similar numbers of quitters and non-quitters were on vareniciline for example. Identification (ID) numbers in the tables are also used to attribute extracts from the data in the narrative text. Most advisers in the total group were male (89%) and from pharmacies in City and Hackney (89%). Sixty-seven percent of advisers were Indian, and 11% were from other black and minority ethnic (BME) groups, with missing ethnicity data for one. Importantly, findings were similar whether the adviser was a pharmacist or trained counter assistant.

VARIABLE	QUITTERS (16)	NON-QUITTERS (16; 6 LTF)	ALL (53; 9 LTF)
FEMALE	3 (19%)	3 (19%)	18 (34%)
AGE (MEDIAN, RANGE)	47 (26-58)	41 (28-59)	41.13 (18-67)
OCCUPATION			
MANAGERIAL AND PROFESSIONAL	2 (13%)	0	5 (9%)
UNEMPLOYED	10 (63%)	10 (63%)	25 (47%)
ROUTINE MANUAL	3 (19%)	3 (19%)	8 (15%)

For peer review only - http://bmjopen.bmj.com/site/about/guidelines.xhtml

**STUDENT**	0	0	1 (2%)
**INTERMEDIATE**	0	2 (13%)	7 (13%)
**RETIRED**	0	0	1 (2%)
**?NO ANSWER**	1 (6%)	4 (25%)	6 (11%)
**ETHNICITY**			
**WHITE BRITISH**	11 (69%)	9 (56%)	30 (57%)
**SOUTH ASIAN**	3 (19%)	3 (19%)	8 (15%)
**OTHER WHITE**	2 (13%)	3 (19%)	10 (19%)
**BLACK AFRICAN**	0	0	1 (2%)
**BLACK CARIBBEAN**	0	0	1 (2%)
**MISSING DATA**	0	1 (6%)	2 (4%)
**CIGARETTES SMOKED**			
**40**	1 (6%)	0	1 (2%)
**30**	2 (13%)	2 (13%)	5 (9%)
**25**	3 (19%)	3 (19%)	8 (15%)
**20**	7 (44%)	9 (56%)	25 (47%)
**15**	1 (6%)	2 (13%)	5 (9%)
**<15**	2 (13%)	1 (6%)	9 (17%)
**PCT**			
**TOWER HAMLETS**	2 (13%)	5	12 (23%)
**CITY AND HACKNEY**	14 (88%)	10	38 (72%)
**NEWHAM**	0	0	3 (6%)

Table 1: Smoker demographic and smoking data summaries for total number sampled and for the matched pair groups (LTF = lost to follow-up).

For peer review only - http://bmjopen.bmj.com/site/about/guidelines.xhtml

Table 2: Summary information for the 16 matched pairs of smokers.

Themes

Thematic analysis reached saturation, and revealed three overarching themes in the data:

- Negotiating the smoker-adviser relationship
- The roles of the adviser and the smoker in the quit attempt
- Smoker and adviser misalignment on reasons for smoking, relapsing and quitting

Table 3 is a quantitative overview of these themes and selected subthemes, comparing quitters and non-quitters (omitted themes are more relevant to our conversation analysis of the data, being reported separately). We follow this with a narrative description of themes; extracts are identified by smoker (P), adviser (A) and consultation week (W) while the quantifiers most, common or many, occasionally or some, and a few or rarely, refer to a theme occurrence of 75%+, 50-74%, 25-49% and less than 25%.

From the quantitative data we determined that the core set of strategies used by advisers (occurring in 50% or more of smokers) were: praise; biomedical talk and advice; mention of the side effects of medication; collaborative talk; the use of props and strategies to cope with the urge to smoke; and the use of monitoring and surveillance talk. Significantly, explicit use of motivational talk is not included in this core set.

Further strategies formed the core set in eventual quitters: talk on the importance of support; open door talk; the need for willpower. Managing expectations formed part of the core set for non-quitters only.

For peer review only

For peer review only - http://bmjopen.bmj.com/site/about/guidelines.xhtml

CODE	NUMBER OF QUITTERS WITH CODE* (% OUT OF n=16)	NUMBER OF NON-QUITTERS WITH CODE* (% OUT OF n=16)	NUMBER OF CONSULTATIONS WITH CODE (%)		TOTAL NUMBER OF OCCURRENCES OVERALL	MEAN FREQUENCY OF MENTIONS IN CONSULTATIONS IN WHICH CODE APPEARS
			QUITTERS* OUT OF 30 CONSULTATIONS, n (%)	NON-QUITTERS* OUT OF 29 CONSULTATIONS n (%)	QUITTERS*. NON-QUITTERS*	QUITTERS*. NON-QUITTERS*
**Negotiating the smoker-adviser relationship**						
Lifeworld talk	10 (63%)	9 (56%)	19 (63%)	18 (62%)	75,45	3.95, 2.5
Adviser being non-judgmental	4 (25%)	3 (19%)	6 (20%)	5 (17%)	9, 10	1.50, 2.00
Adviser praise	10 (63%)	9 (56%)	22 (73%)	20 (69%)	64, 53	2.91, 2.65
**The roles of the adviser and the smoker in the quit attempt**						
Receiving biomedical information and advice	14 (88%)	11 (69%)	25 (83%)	16 (55%)	106, 48	4.24, 3.00
Importance of support	8 (50%)	5 (31%)	14 (47%)	6 (21%)	31, 13	2.21, 2.17
Mentioning side effects of medication	9 (56%)	8 (50%)	12 (40%)	11 (38%)	34, 26	2.83, 2.36
Adviser mentioning relapse	4 (25%)	3 (19%)	9 (30%)	6 (21%)	12, 6	1.33, 1.00
Motivational talk	5 (31%)	5 (31%)	15 (50%)	12 (41%)	37, 34	2.47, 2.83
Confidence in being able to quit	3 (19%)	1 (6%)	8 (27%)	2 (7%)	8, 3	1.00, 1.50
Need for willpower	8 (50%)	7 (44%)	9 (30%)	13 (45%)	17, 18	1.89, 1.38
Collaborative talk (e.g. 'we')	10 (63%)	8 (50%)	20 (67%)	15 (52%)	67, 51	3.35, 3.40

For peer review only - http://bmjopen.bmj.com/site/about/guidelines.xhtml

Ownership of the quit	5 (31%)	4 (24%)	8 (27%)	5 (17%)	16, 6	2.00, 1.20
Managing smoker expectations of the programme	5 (31%)	9 (56%)	9 (30%)	11 (38%)	19, 30	2.11, 2.73
The use of quit props and strategies	11 (69%)	10 (63%)	22 (73%)	22 (76%)	73, 39	3.32, 1.77
'Open door' talk (invitations to informal consultations between the formal ones)	9 (56%)	7 (44%)	10 (33%)	8 (28%)	15, 12	1.50, 1.50
Monitoring and surveillance	14 (88%)	15 (94%)	21 (70%)	17 (59%)	38, 38	1.81, 2.24
**Smoker and adviser misalignment on reasons for smoking, relapsing and quitting**						
Smoker explanations for smoking						
Stress	6 (38%)	9 (56%)	8 (27%)	13 (45%)	19, 17	2.38, 1.31
Social factors	4 (25%)	3 (19%)	6 (20%)	4 (14%)	8, 6	1.33, 1.50
Financial reasons	5 (31%)	5 (31%)	5 (17%)	6 (21%)	6, 8	1.20, 1.33
Health problem	8 (50%)	11 (69%)	8 (27%)	13 (45%)	19, 32	2.38, 2.46
Smoker explanations for wanting to quit						
Quitting for families (including children)	5 (31%)	3 (19%)	6 (20%)	3 (10%)	15, 3	2.50, 1.00
Obligations to self and others	2 (13%)	1 (6%)	4 (13%)	1 (3%)	6, 1	1.50, 1.00
Negative feelings towards smoking	1 (6%)	3 (19%)	1 (3%)	5 (17%)	1, 5	1.00, 1.00
Negative impact of smoking on appearance (e.g. Ageing effects) or identity (e.g. Smelly)	1 (6%)	3 (19%)	2 (7%)	5 (17%)	2, 8	1.00, 1.60
Adviser-suggested motivator for smoker to quit						
Financial	4 (25%)	6 (38%)	10 (33%)	8 (28$)	14, 9	1.40, 1.13

For peer review only - http://bmjopen.bmj.com/site/about/guidelines.xhtml

Health scare tactics	4 (25%)	3 (19%)	5 (17%)	3 (10%)	8, 5	1.60, 1.67

Table 3: Quantitative comparisons between quitters and non-quitters in: a) number of consultations overall in which each code was identified, b) total number of occurrences in all consultations, c) relative frequency with which codes were used in individual consultations. Themes shown here have been selected from a larger pool for this paper; those more relevant to our subsequent conversation analysis have been excluded.

For peer review only

For peer review only - http://bmjopen.bmj.com/site/about/guidelines.xhtml

1. *Negotiating the smoker-adviser relationship*

Lifeworld talk was common across the groups, though occurring in slightly more eventual quitters and with more occurrences within their consultations. This is talk about a person's experience of life with all its contextual and situational nuances.[32-35] Through lifeworld talk, the advisers gained a shared understanding with the smoker of the context of the smoker's quit attempt and the individual difficulties they faced. The adviser could then discuss approaches to quitting that took into account such things as the stress that the smoker in the following extract describes.

.

> A: So whilst you're on Champix and you left work and you're a bit stressed did you say?
> S: Yeah, because there's many changes in the work you remember when you told me in the beginning, like try to, don't change the work and the things. But they change our store manager, my manager is leaving. The work we used to do, six persons now going to be in two persons, oh my god. It's just,
> A: So you're a bit more stressed at work.
> S: Terrible. It's terrible.
> A: So you came home and you're a bit stressed still, so you felt like you wanted a cigarette? But you didn't have a cigarette? (P807-A81-W4, quit)

The advisers made it clear to around one quarter of smokers that they were not going to judge them for any lapses and failures, as a way to motivate them to continue the programme, illustrated in the following extract. This is in line with good practice.

> A: Fantastic. So again, my idea is to mentor you and support you and that's what it's about, yeah? I'm not here to tell you off. It's about where you're finding it hard and what we need to work together to find a solution for you. (P107-A1-W1, quit)

Praise was common across groups, but with marked qualitative differences. Quitters received limited and brief praise. By contrast, eventual non-quitters were given extensive (extreme formulation[36,37]) praise even when they had not achieved the NCSCT-recommended 'not a puff', as below.

> S: Well, it's actually helped a lot, so I'm only smoking around ten.
> A: Ten plus? Ten to fifteen?
> S: Ten plus.
> A: That's fantastic. In the space of six, seven days you've cut down by over half. (P4-A1-W2, not quit)

2. *The roles of the adviser and the smoker in the quit attempt*

Biomedical information and advice was imparted across the groups but quitter consultations were most heavy with it. This may mean they were more open to biomedical talk or that the adviser got an impression that they were, or it may be an incidental finding. Side effects of the medication and withdrawal symptoms from stopping smoking, including weight gain, were discussed in many smokers. The possibility of relapse was discussed with only 25% of quitters and 19% of non-quitters. There was some compensation in the degree to which the use of strategies to deal with cravings (hence *risk* of relapse) was commonly encouraged (see extract later in this section).

Advisers made attempts to manage many of the eventual non-quitters' expectations of the programme as well as emphasising their required commitment. Both strategies were undertaken only occasionally with quitters. Many quitters but only some non-quitters were told of the importance of advisers' counselling support. Advisers' motivational talk was occasional in both

For peer review only - http://bmjopen.bmj.com/site/about/guidelines.xhtml

groups, and did not part of the core set of strategies and advisers only rarely asked for (and therefore got) expressions of confidence in being able to quit (3 quitters over 8 consultations vs 1 non-quitter over 2 consultations), a specific element of motivational interviewing. The need for the smoker to draw on their own willpower to quit was considered in most quitters but only some non-quitters, and the necessary balance between adviser support and more personal willpower or motivation was only discussed with one non-quitter (in the following extract) and one quitter.

> S: I've got a photograph of me taken when I was in intensive care in hospital, I should take a look at that every time I want a cigarette.
> A: Perfect! You need some motivation.
> S: I don't want to end up like that again.
> A: Exactly, you need that motivation. And if there's anyone else that can help you also…
> S: My mum, yeah, she's very supportive.
> A: Sure. You need that support as well. But you've got us as well because you'll be coming here every week. (P806-A81-W1, not quit)

Collaborative talk (such as the use of the phrase "we will succeed"), a supportive stratagem, was common in both groups. The related topic 'smoker's ownership of the quit' was rarely mentioned in non-quitter consultations and only slightly more so in quitters'.. Some messages were implied rather than explicit. The use of props and strategies to deal with cravings requires some ownership and self-management of the quit and was recommended at least once in a majority of smokers and consultations, though more frequently discussed *within* quitter consultations. Many different props and strategies were suggested, with the following only one example.

> A: I want you to make sure you start off in the morning; you've got your bottle of 1.5 litres to 2 litres. Your aim, you start in the morning, you finish at the end of the day. Every time you want a cigarette, you're going to drink the water. You're going to complete it. (P101-A11-W1, not quit).

The advisers invited many eventual non-quitters to drop into the pharmacy for support and advice any time the shop was open, a particular advantage that pharmacies have over other healthcare settings. Such 'open door' talk, offering a more informal variant of adviser support, was said to fewer eventual non-quitters and was less successful for these.

In most smokers, the CO monitor was depicted by the adviser as a monitoring or surveillance tool that was used to check the smoker's progress and even catch the smoker out:

> A: Well, it's good that you're here, we will help you in any way we can. What I ask you every week is if I can take a reading from you. This is called a CO monitor and this machine measures the amount of carbon monoxide inside of your system. And I call this my lie detector. I'm not going to say that you're going to lie to me, but if you do come in and I say have you had any cigarettes, and you say no, I haven't had any, this will tell me the truth. (P7-A1-W1, quit)

3. *Smoker and adviser misalignment on reasons for smoking, relapsing and quitting*

An actual health problem or worry about getting one was cited as reason to quit by more than half the smokers and was invoked slightly more commonly by non-quitters. There was an inverse relationship between this and adviser use of health scare tactics to motivate the smoker, though these tactics were relatively uncommon. Financial reasons for wanting to quit were cited by just under one third of smokers. However the advisers tried to motivate the smokers with talk of

For peer review only - http://bmjopen.bmj.com/site/about/guidelines.xhtml

financial savings on 23 separate occasions. Misalignment is demonstrated in the following extract for example.

> S: I mean I don't want to die. I don't want to have a stroke.
> A: So that's your main impulse now and more likely you'll succeed because of that.
> S: I don't want to end up like that…
> A: The other reasons why maybe you feel I'll give up now, maybe save a bit of money or whatever before…
> S: That's the backburner, that's nothing to do…
> A: Exactly. Was that the reasons before?
> S: No.
> A: What were the reasons before that you wanted to stop smoking?
> S: Smell; I don't like the smell. I've got this thing, when I eat, people smoke around me, I don't like it, I don't like the smell of it when I'm eating. People light one up when you're having something to eat and oh, it's disgusting, I don't like the smell. (P806-A81-W1, not quit)

Quitters were twice as likely to mention their families (including children) as motivation though under a third did so. Other reasons for smoking were cited relatively infrequently. Notably the misalignment or mismatch between smoker declaration of health as motivation to quit and adviser comments on health problems caused by smoking was more likely in non-quitters, and advisers were also less likely to mention motivational factors at all to eventual non-quitters. In quitters' consultations, advisers were likely to mention motivators, especially financial, even when the smoker did not.

Discussion

We applied rigorous and systematic analytical methods to a sample of audiorecordings of real world community pharmacy adviser-smoker NHS Stop Smoking consultations with the premise that the community pharmacy Stop Smoking programme should be person-centred. Specifically, we explored associations of thematic patterns in the verbal interactions with 4-week quit success. Although there are a number of ethnographic studies of client-pharmacy staff communication processes or interactions[24, 52-74,77] there is none on stopping smoking in the community. Our overall findings accord with those of Pilnick's study[54-56] in a UK hospital paediatric outpatient clinic pharmacy, the most comparable study to ours, which focussed on advice-giving in the counselling role, but we provide rich new data. We found many examples of good practice and significant evidence of lifeworld talk which enables person-centred support, but also differences between the quitter and non-quitter consultations that had the potential to affect smoker retention in the Stop Smoking programme and quit outcome. The core set of strategies used was weighted towards the practical and the medical (the 'voice of medicine',[33] see below), with psychologically and emotionally supportive strategies concentrated in the quitter compared with the non-quitter core set. It was striking that even when strategies that accord with best practice were used, this was in inconsistent ways that sometimes had the potential to create misalignment between smoker and adviser. In the case of quit motivators, this misalignment could be determined from simple comparison of the mention of specific factors as clear from our example extract, but in other cases discussed below the misalignment only became apparent from detailed examination of the data. There were also some clinically potentially significant omissions across the data. For example talk about relapse was rare, though there was some compensation in the degree to which the use of strategies to deal with cravings was frequently encouraged and the biomedical problems of quitting often explored.

For peer review only - http://bmjopen.bmj.com/site/about/guidelines.xhtml

That lifeworld talk was common across the two groups is in itself a positive finding. Mishler,[33] applying the Habermas theory of Communicative Action[43] to medical encounters, itself derived from Husserl's phenomenological concept of consciousness and subjectivity,[34] showed how a bias towards the voice of the patient's lifeworld rather than the voice of medicine enabled clinicians to heed and work with patients' nuanced and contextualised accounts of their presentations and management of their health. Recommendations, advice and treatments are more likely to work if their fit to the patient's lifeworld has been taken into account.[35] The occurrence of lifeworld talk in our data suggests that the community setting and the advisers' social familiarity with the smokers as customers enables such smoker-centred care, in a way that is potentially greater than is possible in GP consultations. This supports other research suggesting that community pharmacies are ideally placed for smoking cessation and other health behaviour change tasks.[10-13] However the considerable lifeworld talk did not always result in a successful quit. The voice of medicine, that is the use of biomedical talk and advice, was also frequent in both quitters and non-quitters; this was appropriate given the pharmacy staff member's role as *adviser*.

As a less positive finding we found several examples of communication patterns that could potentially create confusion or misalignment in service users. Advisers tended to offer casual 'open door' drop-in support between consultations more often to eventual non-quitters, perhaps because they perceived they needed more support, but micro-detailed inspection of the data (not reported here) suggests this simply confused smokers as to when they were meant to attend. Praise and willpower talk were common in both quitters and non-quitters but the way they were communicated was often poorly matched to smoker effort. There were further striking examples of the potential for misalignment in the way advisers drew on financial and health benefits as motivators even when other reasons for quitting were cited by the smokers. Although this may sometimes represent a misalignment between smoker and adviser it may in other cases mean that health scare or financial motivator talk by advisers obviated the need for the smoker to mention these.

Misalignments push interactants 'out of sync' with each other, making it hard for them to achieve a common goal. Nyugen considers how misalignments in pharmacy counselling need to be delicately managed in order not to affect the tenor of the rest of the conversation.[68] Misalignments were noted by Pilnick[54-56] as problematizing the adviser's advice giving but we have shown how they create issues much earlier in the adviser's cognitive development of what that advice might be as well as how their counselling support should be configured.

We found misalignments to be more common in non-quitters. Given that their consultations contained considerable lifeworld information, and considering the inconsistencies in how such information was used, it appeared that advisers used lifeworld talk to inform judgments about likely smoker quit success. This then compromised effective use of such talk in non-quitters disproportionately – and less exploration of the nuances by advisers - in ways that might have affected quit outcome. This could explain why a smoker's sharing of their lifeworld was often not associated with quit success. This interpretation of the data is supported by our separate analysis of adviser interviews.[76] There were strong indications that such pre-judgements affected praise and willpower talk and the core set of strategies used in each group. Ironically, since the modified strategies, though well meaning, led to misalignment of adviser and eventual non-quitter in particular, they may have increased the likelihood that a quit attempt would fail.

Our data also show that the advisers drew on a number of sometimes competing strategies to induce a quit, including combining reassurance of a non-judgmental approach with the use of the CO monitor as a surveillance tool. While this seems to have been successful, biomedical talk and

For peer review only - http://bmjopen.bmj.com/site/about/guidelines.xhtml

advice, talk of being non-judgmental, monitoring and surveillance, and the advisers' use of authority and expertise to prejudge likely smoker success, are all premised on a power differential between adviser and smoker and may therefore appear to sit in dialectical tension with the development of shared understandings and social familiarity from the community setting. Social familiarity and power differentials are not mutually exclusive but they are less likely to co-exist the greater the difference in social status between two interactants. Thus the pharmacy community setting provides particular communication issues that are less likely to occur in encounters with doctors and medical specialists. Social familiarity is shown in our data to have the potential to enhance quit success and power differentials to compromise this, and the best outcomes might be achieved when advisers strike a balance between the two, optimising the benefits of the community pharmacy setting. Figure 1 demonstrates this. Considering recommendations for pharmacy adviser smoking cessation care, we found more person centred care, drawing motivators from clients, and with use of certain techniques such as consistency in the use of the not a puff rule that avoided the problems of over-praising are more likely to result in a successful quit.

Strengths and limitations

Our study contributes to current knowledge and practice with novel data from a pharmacy service that has not previously been subject to ethnographic study. As this was a qualitative study we considered data from a small and varied sample of participating staff members, smokers and community pharmacies, adding to richness of the findings. Since we were comparing two groups, we matched them on relevant variables to increase the dependability of our analysis. We achieved theme saturation within this matched pair design, with adequate agreement between coders, group data sessions and consideration of negative cases in the data adding to credibility and dependability of findings. Themes reported in this analysis correspond with some of the themes developed separately from semi-structured qualitative interviews with advisers and smokers from the same study.[77] Our study has enabled us to identify key elements of talk and can be used to strengthen consultations and make them more effective.

Our study also has limitations. We were not successful in recruiting pharmacy chains and make no claims to transferability of the findings to other pharmacies, particularly those in more affluent areas. Further, it may not be possible to extrapolate findings from these consultations, which were undertaken in a private room, to over the counter community pharmacy interactions. In collecting naturalistic qualitative data, our aim was rather to provide in depth exploration of communication practices. This was usefully achieved. A number of contextual and demographic variables are known to affect pharmacist-smoker communication and quit successes.[78] We were unable to explore these in our small sample. Participants were not blinded to the nature of the research and had a potential vested interest in successful quits, creating a possible performance effect. However the unobtrusive data collection method will have reduced this risk with smokers and the effect would have been the same for quitters and non-quitters. There may have been an intrinsic difference between the quitters and non-quitters before they even entered the consultation room and our study was not designed to explore this. For example, although baseline characteristics known to be related to quitting, such as gender and age were taken into account we did not systematically collect data on other potentially significant variables such as number of previous quit attempts or medication use. Our thematic analysis suggests this would have been useful. The quitters may have been successful irrespective of consultation features; the availability of support itself may be sufficient.[79]

For peer review only - http://bmjopen.bmj.com/site/about/guidelines.xhtml

Clinical implications

Community pharmacy Stop Smoking advisers may need to reflect on the inconsistencies in their delivery of and the content of the consultations. They should begin with consideration of their use of the considerable lifeworld talk generated by smokers through their social familiarity with the pharmacy advisers. Lifeworld talk should be used to modify adviser counselling to suit key external psychosocial factors, namely the *context* within which the quit attempt is made and the related *motivators* to quitting, but our data suggest that advisers may modify the consultation to suit their assumptions about internal psychological factors such as the sm*oker's capacity to quit or motivation for quitting* (factors that we consider separately in a companion paper).[77] In this way advisers sometimes develop the consultation in ways that, while well-meaningly intended, run the risk of being counter-productive. For smokers judged by them as less likely to quit this may include inappropriately overemphasising some support strategies (such as praise, managing expectations) and underplaying others (such as the need for willpower and the importance of adviser support). Our data illustrate the potential for misalignment that this creates. Moreover, the exaggerated praise we saw in non-quitter reductions in smoking conflicts with the 'not a puff' rule recommended by the NCSCT. Advisers should use findings from our study to clarify where they might usefully capitalise on the social closeness they seem to share with the smokers they see, with more appropriate use of lifeworld talk and more use of smoker-declared motivators during motivational interviewing.

Recommendations for research

Our study only considers short-term outcomes, something we are addressing with further research. The analyses have been used to inform the development and content of our training intervention for pharmacy Stop Smoking advisers, evaluated in a cluster randomised trial (grant number RP-PG-0609-10181). We are currently exploring the micro-detail of the talk in the consultations using conversation analysis with a view to further dissemination and use. Our study could be replicated in less disadvantaged areas or in specific minority groups.

4487 words

Acknowledgments The authors thank participants and their community pharmacies. This paper presents independent research funded by the National Institute for Health Research (NIHR) under its Programme Grants for Applied Research Programme. The views expressed are those of the authors and not necessarily those of the NHS, the NIHR or the Department of Health.

Contributors RW conceived the study. CR undertook the analysis and wrote the manuscript. RS, LA and VM were involved in data collection. RS, LS and RW contributed to the data analysis and interpretation. VM, LS, LA, EE, CG, ST, SE and RW were involved in revising the article critically and contributing important intellectual content. RW is the guarantor.

Competing interests None.

Funding: NIHR Programme grant RP-PG-0609-10181

For peer review only - http://bmjopen.bmj.com/site/about/guidelines.xhtml

References

1. Gidman W, Ward P, McGregor L. Understanding public trust in services provided by community pharmacists relative to those provided by general practitioners: a qualitative study. *BMJ Open* 2012;2:3 e000939 doi:10.1136/bmjopen-2012-000939
2. The Royal Pharmaceutical Society of Great Britain. *Pharmacy in a New Age: The New Horizon.* London: The Royal Pharmaceutical Society of Great Britain, 2006.
3. Department of Health. *Pharmacy in England: Building on strengths – delivering the future.* London: The Stationery Office, 2008.
4. Department of Health. *Speech by the Rt Hon Alan Johnson MP, Secretary of State for Health NHS Confederation Primary Care Network,* 2008 webarchive.nationalarchives.gov.uk/+/www.dh.gov.uk/en/MediaCentre/Speeches/Speecheslist/DH_083369 (accessed 21 December 2015)
5. Royal Pharmaceutical Society. *The Changing Face of Pharmacy,* https://www.rpharms.com/public-affairs-pdfs/rps-changing-face-of-pharmacy-booklet.pdf (accessed 9 January 2016)
6. NHS England. *Improving health and patient care through community pharmacy - a call to action,* 2013. http://www.england.nhs.uk/wp-content/uploads/2013/12/community-pharmacycta.pdf (accessed 24 Feb 2015)
7. NHS Employers. *Pharmacy-based stop smoking services: optimising commissioning*. London: NHS Employers, 2009.
8. Pharmaceutical Services Negotiating Committee, *The community pharmacy: A guide for general practitioners and practice staff.* July 2013.
9. NHS Confederation. *Health on the high street. Rethinking the role of community pharmacy,* London, 2013. www.nhsconfed.org/Publications/reports/Pages/Health-on-high-street-rethinking-community-pharmacy.aspx (accessed 24 Feb 2015)
10. National Pharmacy Association. *Pharmacy Health Trainers Resource*, 2009.
11. Fajemisin F. *Community Pharmacy and Public Health. Solutions for Public Health*, 2013. URL: http://www.sph.nhs.uk/sph-documents/community-pharmacy-and-public-health-finalreport/?searchterm=community pharmacy (accessed 21 Dec 2015)
12. Hametz I, Hodgkiss F, Sheehy C. *Review of the community pharmacy public health service for smoking cessation and emergency hormonal contraception*. Scottish Government, Edinburgh; 2011
13. Todd A, Copeland A, Husband A, Kasim A, Barbra C. The positive pharmacy care law: an area-level analysis of the relationship between community pharmacy distribution, urbanity and social deprivation in England, BMJ Open 2014;4:8 e005764 doi:10.1136/bmjopen-2014-005764
14. Ali A, Wright N, Rae M. Addressing Health Inequalities – A guide for general practitioners. Royal College of General Practitioners Health Inequalities Standing Group. London: RCGP, 2008
15. Lifestyle statistics, Health and Social Care Information Centre. *Statistics on NHS Stop Smoking Services: England, April 2012 – March 2013,* 2013
16. West R, May S, West M, Croghan E, McEwen A. Performance of English stop smoking services in first 10 years: analysis of service monitoring data. *BMJ* 2013;47:f4921.
17. Bartlett EE, Grayson M, Barker R, Levine DM, Golden A, Libber S. The effects of physician communications skills on patient satisfaction; recall, and adherence. *J Chronic Dis.* 1984;37(9–10):755–764
18. Epstein RM, Street RL. The Values and Value of Patient-Centered Care. *Annals of Family Medicine* 2011;9(2):100-103. doi:10.1370/afm.1239

For peer review only - http://bmjopen.bmj.com/site/about/guidelines.xhtml

19. The Health Foundation. *Person-centred care made simple: What everyone should know about person-centred care*, The Health Foundation, London 2014.
20. Department of Health. Equity and Excellence: Liberating the NHS. Department of Health: London, July 2010
21. National Institute for Health and Care Excellence. *Falls in older people: assessing risk and prevention. NICE guidelines [CG161],* June 2013
22. Royal Pharmaceutical Society Wales. *Your Care, Your Medicines: Pharmacy at the heart of patient-centred care*, 2015
23. Greenhill N, Anderson C, Avery A, Pilnick A. Analysis of pharmacist-patient communication using the Calgary-Cambridge guide. *Patient Education and Counseling* 2001; (83): 423-431
24. Salter C, Holland R, Harvey I, Henwood K. 'I haven't even phoned my doctor yet'. The advice giving role of the pharmacist during consultations for medication review with patients aged 80 or more: qualitative disclosure analysis. *BMJ* 2007; May 26;334(7603):1101
25. Department for Communities and Local Government, *English indices of deprivation 2015*, Department for Communities and Local Government, London, 2015
26. West R, Hajek P, Stead L, Stapleton J. Outcome criteria in smoking cessation trials: proposal for a common standard. *Addiction* 2005;100: 299–303. doi: 10.1111/j.1360-0443.2004.00995.x
27. National Institute for Health and Care Excellence. *Training in smoking prevention and cessation.* NICE, 2015.
28. Bauld L, Chesterman J, Ferguson J, Judge K. A comparison of the effectiveness of group-based and pharmacy-led smoking cessation treatment in Glasgow. *Addiction* 2009; 104: 308-16
29. Bauld L, Debbie F. *Local Stop Smoking Services Service and delivery guidance 2014*, 2014 National Centre for Smoking Cessation and Training
30. Braun V, Clarke V. *Thematic analysis.* In: Cooper, H., ed. (2012) APA Handbook of Research Methods in Psychology. (Vol. 2) Washington, DC: American Psychological Association).
31. Rivas C. *Coding qualitative data*. In, Seale, Clive (ed.) Researching Society and Culture. Third Edition. London, GB, Sage
32. Hemingway A. Lifeworld-led care: is it relevant for well-being and the fifth wave of public health action? *Int J Qual Studies on Health and Well-being* 2011;6 (4), Article ID 10364
33. Mushier EG. *The discourse of medicine. The dialectics of medical interviews*, Ablex, Norwood, NJ, 1984
34. Husserl E. *The crisis of European science and transcendental phenomenology*, Northwestern University Press, Evanston, Ill, USA, 1970
35. Barry CA, Stevenson FA, Britten N, Barber N, Bradley CP. Giving a voice to the lifeworld. More humane, more effective medical care? A qualitative study of doctor-patient communication in general practice. *Soc Sci Med* 2001; 53, 487–505
36. Edwards D. Extreme case formulations: softeners, investment, and doing nonliteral. *Research on Language and Social Interaction* 2000;33(4), 347–373
37. Pomerantz A. Extreme case formulations: A way of legitimizing claims. *Human Studies* 1986; 9, 219–229
38. Goffman E. *Interaction Ritual: Essays on face-to-face behavior.* (New York: Doubleday Anchor, 1967) (London: Allen Lane, 1972)
39. Britten N, Stevenson FA, Barry CA, Barber N, Bradley CP. Misunderstandings in prescribing decisions in general practice: qualitative study *BMJ* 2000; 320 :484.
40. Trinkaus Zagzebski L. Epistemic authority: a theory of trust, authority, and autonomy in belief, Oxford University Press, Oxford, 2012

For peer review only - http://bmjopen.bmj.com/site/about/guidelines.xhtml

41. Seale C, Rivas C, Kelly M. The challenge of communication in interpreted consultations in diabetes care: a mixed methods study. *The British Journal of General Practice* 2013;63(607):e125-e133. doi:10.3399/bjgp13X663082.
42. Webb H. ‘I've put weight on cos I've bin inactive, cos I've ’ad me knee done’: moral work in the obesity clinic. *Sociology of Health and Illness* 2009; 31 (6), 854–871
43. Habermas J. *The theory of communicative action, Reason and the rationalization of society,* vol. 1. London: Heinemann, 1984
44. Rollnick S, Miller WR. What is Motivational Interviewing? *Behavioural and Cognitive Psychotherapy* 1995;23:325-334
45. Heritage J, Lindstrom A. Motherhood, medicine, and morality: scenes from a medical encounter. *Research on Language and Social Interaction* 1998; 31, 397–438
46. Heritage J, Robinson JD. *Accounting for the visit: giving reasons for seeking medical care*. In: J. Heritage, D.W. Maynard (Eds.), Communication in medical care: Interaction between primary care physicians and patients, Cambridge University Press, Cambridge, 2006, pp. 48–85
47. McMullen LM, Herman J. Women's accounts of their decision to quit taking antidepressants. *Qualitative Health Research* 2009;19 (11), 1569–15
48. Peräkylä A. Authority and accountability: the delivery of diagnosis in primary health care. *Social Psychology Quarterly* 1998;61 (4), 301–320
49. Roberts C, Sarangi S, Moss B. Presentation of self and symptoms in primary care consultations involving patients from non-English speaking backgrounds. *Communication & Medicine* 2004; 1 (2),159–169
50. Stephens C, Breheny M. Menopause and the virtuous woman: the importance of the moral order in accounting for medical decision making. *Health* 2008; 12, 7–24
51. Seale C, Rivas C, Al-Sarraj H, Webb S, Kelly M. Moral mediation in interpreted health care consultations, *Social Science & Medicine* 2013; 98, 141-148, ISSN 0277-9536, http://dx.doi.org/10.1016/j.socscimed.2013.09.014.
52. Cavacoa A, Roter D. Pharmaceutical consultations in community pharmacies: utility of the Roter Interaction Analysis System to study pharmacist–patient communication. *IJPP* 2010, 18: 141–148
53. Shah B, Chewning B. Conceptualizing and measuring pharmacist-patient communication: a review of published studies. *Res Soc Admin Pharm* 2006; 2: 153–185
54. Pilnick A. The interactional organization of pharmacist consultations in a hospital setting: a putative structure. *J Pragmatics* 2001; 33, 1927–1945
55. Pilnick A. “Patient counseling” by pharmacists: advice, information, or instruction? *Sociol Quart* 1999; 40, 613–622
56. Pilnick A. "Patient counselling" by pharmacists: four approaches to the delivery of counselling sequences and their interactional reception. *Soc Sci Med* 2003; 56, 835–849.
57. Garner M, Watson MC. Using linguistic analysis to explore medicine counter assistants' communication during consultations for nonprescription medicines. *Patient Educ Couns* 2007; 65,51–57
58. Stevenson FA. Achieving visibility? Use of non-verbal communication in interactions between patients and pharmacists who do not share a common language. *Sociology of Health & Illness* 2014; 36(5), 1467. DOI:10.1111
59. Stevenson FA, Leontowitsch M, Duggan C. Over-the-counter medicines: professional expertise and consumer discourses. *Sociol Health Illn* 2008;30,913–928
60. Leontowitsch M, Stevenson F, Nazareth I, Duggan C.‘At the moment it is just a couple of eccentrics doing it’: concordance in day-to-day practice. *Int J Pharm Pract* 2005; 13, 273–280

For peer review only - http://bmjopen.bmj.com/site/about/guidelines.xhtml

61. Evans SW, John DN. A preliminary investigation of the interactions between UK and US community pharmacists and their prescription clients. *Int J of Pharmacy Practice* 1995; 3: 157–162. doi: 10.1111/j.2042-7174.1995.tb00810.x
62. Pelicano-Romano J, Neves MR, Amado A, Cavaco AM. Do community pharmacists actively engage elderly patients in the dialogue? Results from pharmaceutical care consultations. *Health Expectations* 2015, 18: 1721–1734. doi: 10.1111/hex.12165
63. Chen J, Britten N. 'Strong medicine': an analysis of pharmacist consultations in primary care. *Fam Pract* 2000;17, 480–483
64. Babalola O, Erhun WO. Pharmacist medication history interview of elderly patients in a teaching hospital in Nigeria. *Int J Pharm Pract* 2001;9, 97–103
65. Salter C. Compliance and concordance during domiciliary medication review involving pharmacists and older people. Sociol Health Illn, 32 (2010), pp. 21–36
66. Hargie OD, Morrow NC, Woodman C. Pharmacists' evaluation of key communication skills in practice. *Patient Educ Couns* 2000;39, 61–70
67. Dyck A, Deschamps M, Taylor J. Pharmacists' discussions of medication side effects: a descriptive study. *Patient Educ Couns* 2005; 56, 21–27
68. Nguyen HT. Boundary and alignment in multiparty conflict talk. *J Pragmatics* 2011; 43, 1755–1771
69. Bissell P, Ward P, Noyce PR. Appropriateness measurement: application to advice-giving in community pharmacies. *Soc Sci Med* 2000;51, 343–359
70. Skoglund P, Isacson D, Kjellgren DI. Analgesic medication–communication at pharmacies. *Patient Educ Couns* 2003; 51,155–161
71. Montgomery AT, Kettis Lindblad A, Eddby P, Soderlund E, Tully MP, Kalvemark Sporrong S. Counselling behaviour and content in a pharmaceutical care service in Swedish community pharmacies. *Pharm World Sci* 2010;32,455–463
72. Watermeyer J,Penn C. "Tell me so I know you understand": pharmacists' verification of patients' comprehension of antiretroviral dosage instructions in a cross-cultural context. *Patient Educ Couns* 2009;75, 205–213
73. Watermeyer J. "Now here come the pills that are going to save your life": pharmacists' discussions of antiretroviral drugs in a context of life and death. *AIDS Care* 2011; 23,807–813
74. Watermeyer J, Penn C. The organization of pharmacist-patient interactions in an HIV/aids clinic. *J Pragmatics* 2009, 41,2053–2071
75. Ylanne V, John DN. Roles of medicines counter assistants in advice giving in community pharmacies: a discourse analysis. *Pharm World Sci* 2008;30, 199–2077
76. Sohanpal R, Rivas C, Steed L., MacNeill V, Kuan V, Edwards E, Griffiths C, Eldridge S, Taylor S, Walton R. Understanding recruitment and retention in the NHS community pharmacy stop smoking service: perceptions of smoking cessation advisers. *BMJ Open* (submitted).
77. Paluck EC, Green LW, Frankish CJ, Fielding DW, Haverkamp B. Assessment of communication barriers in community pharmacies. *Eval Health Prof* 2003 26: 380
78. Lancaster T, Stead LF. Individual behavioural counselling for smoking cessation. *Cochrane Database of Systematic Reviews* 2005, Issue 2. Art. No.: CD001292. DOI: 10.1002/14651858.CD001292.pub2.

For peer review only - http://bmjopen.bmj.com/site/about/guidelines.xhtml

Figure 1: Tentative model of misalignment in the community pharmacy NHS Stop Smoking service consultation.

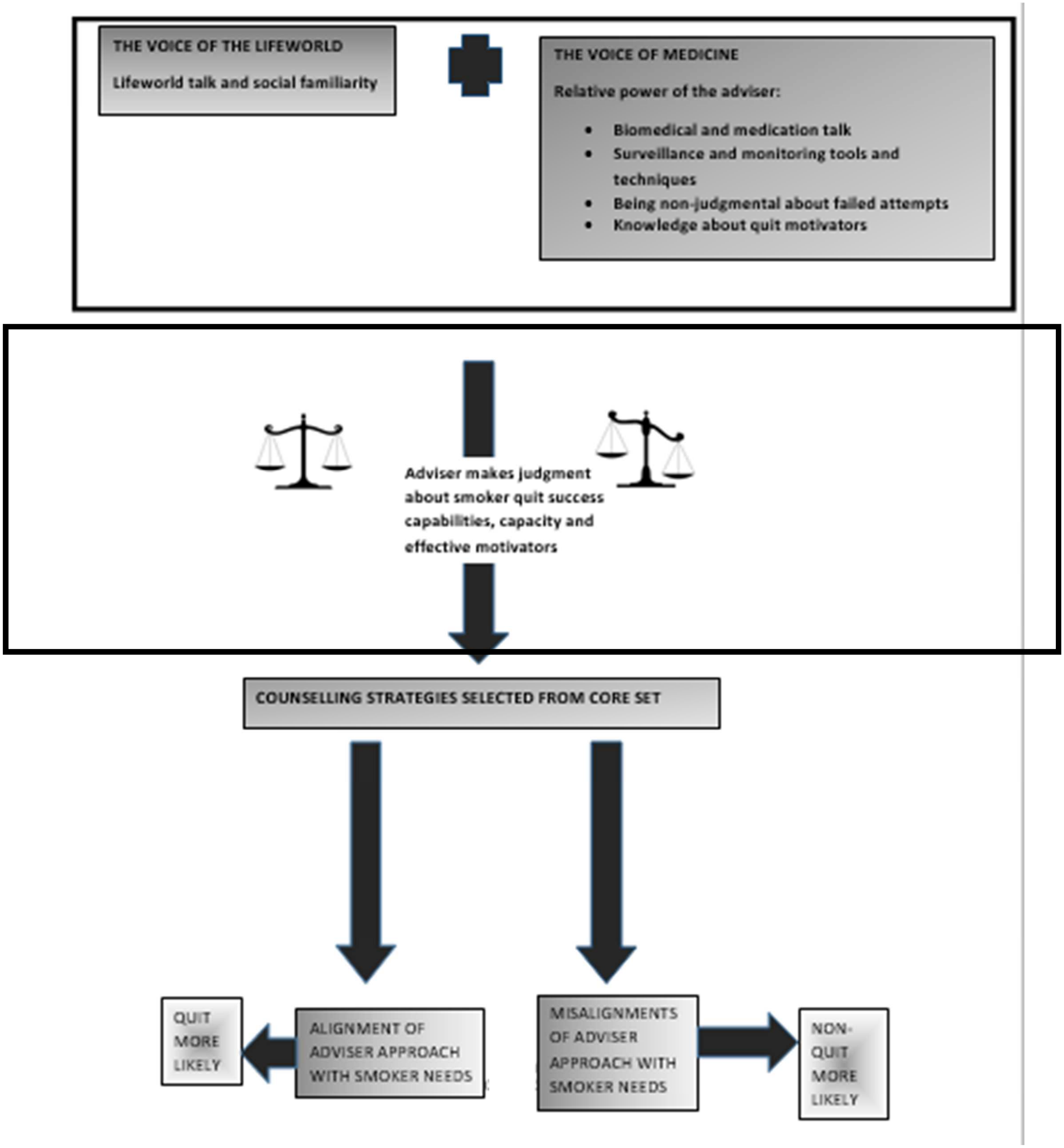

BMJ Open

Determining counselling communication strategies associated with successful quits in the National Health Service community pharmacy Stop Smoking programme: a focused ethnography using recorded consultations

Journal:	*BMJ Open*
Manuscript ID	bmjopen-2016-015664.R1
Article Type:	Research
Date Submitted by the Author:	22-May-2017
Complete List of Authors:	Rivas, Carol; University College London Social Science Research Unit; Queen Marys School of Medicine and Dentistry, Centre for Primary Care and Public Health<br>Sohanpal, Ratna; Queen Marys School of Medicine and Dentistry, Centre for Primary Care and Public Health<br>MacNeill, Virginia; University of Oxford<br>Steed, Liz; Queen Marys School of Medicine and Dentistry, Centre for Primary Care and Public Health<br>Edwards, Elizabeth; Queen Mary University of London, Centre for Primary Care and Public Health<br>Antao, Laurence; Queen Mary University of London<br>Griffiths, Chris; Queen Mary University of London<br>Eldridge, Sandra; Queen Mary University of London, Centre for Primary Care and Public Health<br>Taylor, Stephanie; Queen Mary University of London, Primary Care and Public Health<br>Walton, Robert; Queen Mary University of London
**Primary Subject Heading**:	Smoking and tobacco
Secondary Subject Heading:	Health services research, Public health, Qualitative research, Patient-centred medicine
Keywords:	Smoking cessation, Community Pharmacies, QUALITATIVE RESEARCH, Communication, Focused ethnography

SCHOLARONE™ Manuscripts

Determining counselling communication strategies associated with successful quits in the National Health Service community pharmacy Stop Smoking programme: a focused ethnography using recorded consultations

Carol Rivas[12]

Ratna Sohanpal[2]

Virginia MacNeill[3]

Liz Steed[2]

Elizabeth Edwards[2]

Laurence Antao[2]

Chris Griffiths[2]

Sandra Eldridge[2]

Stephanie Taylor[2]

Robert Walton[2]

[1] SSRU, University College London, UK

[2]Centre for Primary Care and Public Health, Queen Mary University of London, London, UK

[3]University of Oxford, UK

Corresponding author:

Carol Rivas

Senior Researcher (Associate Professor)

SSRU

University College London

London, WC1H 0NR

Email: c.a.rivas@qmul.ac.uk

For peer review only

For peer review only - http://bmjopen.bmj.com/site/about/guidelines.xhtml

ABSTRACT (293/300words)

Objectives: To determine communication strategies associated with smoking cessation in the National Health Service community pharmacy Stop Smoking programme.

Setting: Eleven community pharmacies in three inner east London boroughs.

Participants: Nine Stop Smoking advisers and sixteen pairs of smokers who either quit or did not quit at four weeks, matched on gender, ethnicity, age and smoking intensity.

Method: 1-3 audio-recorded consultations between an adviser and each pair member over 5-6 weeks were analysed using a mixed method approach. First a content analysis was based on deductive coding drawn from a theme-oriented discourse analysis approach and the Roter Interaction Analysis System (RIAS). Core themes were identified through this quantification to explore in detail the qualitative differences and similarities between quitters and non-quitters.

Results: Quantitative analysis revealed advisers used a core set of counselling strategies that privileged the 'voice of medicine' and often omitted explicit motivational interviewing. Smokers tended to quit when these core strategies were augmented by: supportive talk; clear permission for smokers to seek *additional* support from the adviser between consultations; encouragement for smokers to use willpower. The thematic analysis highlighted the choices made by advisers as to which strategies to adopt and the impacts on smokers. The first theme 'Negotiating the smoker-adviser relationship' referred to adviser judgements about the likelihood the smoker would quit. The second theme 'Roles of the adviser and smoker in the quit attempt', focused on advisers' counselling strategies, while the third theme, 'Smoker and adviser misalignment on reasons for smoking, relapsing and quitting' concerned inconsistencies in the implementation of NCSCT training recommendations.

Discussion: Advisers in community pharmacies should use the advantages of their familiarity with smokers to ensure appropriate delivery of patient-centred counselling strategies, and reflect on the impact on their counselling of early judgments of smoker success.

Keywords: Smoking cessation, Community Pharmacies, Qualitative research, Focused ethnography, Communication

For peer review only - http://bmjopen.bmj.com/site/about/guidelines.xhtml

Strengths and limitations of this study

- To our knowledge this is the first study to consider verbal interactions in real world consultations between community pharmacy advisers and smokers in the NHS community pharmacy Stop Smoking programme
- 32 smokers from a larger sample were matched on demographic variables to provide 16 matched pairs of four-week quitters and non-quitters, enhancing confidence in findings.
- The findings may not be transferable to other pharmacies, particularly in less disadvantaged areas, or to over the counter community pharmacy interactions since the consultations took place in dedicated consultation rooms.
- Our study findings are limited to interactions during the consultation, as appropriate to the methods chosen, and we did not systematically explore previous quit attempts and their influence on results.

Introduction

UK community pharmacies have adopted public health and healthy living tasks that were previously the domain of the general practitioner.[1] These 'enhanced services' are embedded in UK health service policy and embraced by pharmaceutical professional bodies.[2-9] Community pharmacies have great potential to provide such health care, particularly for people from disadvantaged groups,[10] given their community setting, long opening hours, accessibility, familiarity and informality.[11-13] Pharmacy enhanced services therefore provide an opportunity to address health inequalities and may free general practice time for patients with more complex problems.[14]

The NHS Stop Smoking programme is one of the most frequently delivered enhanced community pharmacy services in England[15,16] and its effectiveness in 2015/16 measured by smoker self-reported 4-week quit rates was only slightly lower than for general practice (44% vs. 49%).[17] But neither general practice nor community pharmacies achieve the suggested optimal quit rate of 70%,[7] and there is considerable variation in rates within the same setting.[17] Moreover, the proportion of smokers enrolling on the Stop Smoking programme in community pharmacies rather than GP surgeries and other settings has remained stable since the programme began in 2006, falling between 18-21%, for example 20% in 2015/16.[7,17] This is despite government efforts to shift some of the GP workload to them.[7,15]

Enhanced services have necessitated a change in mind set for pharmacy staff with a move towards the person-centred care model currently advocated within health services.[17-26] Pharmacy staff tend to be inadequately trained in this model,[24] and may lack necessary consultation skills,[24,26] which may affect the growth of the Stop Smoking service in pharmacies. We therefore used a 'focused ethnography' approach[27,28] to explore counselling strategies used in consultations between pharmacy Stop Smoking advisers and smokers. Our primary aim was to use the findings to develop an intervention intended to improve both service uptake and quit rates in the community pharmacy Stop Smoking programme, as reported elsewhere.[29]

Methods

Focused ethnography

Using focused ethnography[27,28] we collected and analysed recordings of naturalistic (real world) consultations between pharmacy Stop Smoking advisers and smokers; recordings were made by the advisers themselves for the study. We considered verbal interactions that might affect smoker engagement, maintenance within the pharmacy programme and success in quitting.

For peer review only - http://bmjopen.bmj.com/site/about/guidelines.xhtml

In traditional ethnographic work, researchers immerse themselves in the social worlds of their participants to better understand the ways they interact with these worlds. They then generate textual or visual accounts of this for dissemination.[30] In focused ethnography, field visits are either much shorter, and selectively focussed, or replaced by audio- or video- or photo-recordings which may be undertaken with the researcher absent from the scene, as in our study. This approach is useful for answering a specific research question when traditional ethnography is impractical.[28]

Pharmacies and advisers

Consultations were recorded between November 2013 and May 2014 at community pharmacies contracted by Public Health England to provide the Stop Smoking programme in three inner East London boroughs (Newham, Tower Hamlets, the City and Hackney). These areas are economically disadvantaged compared with other areas of London and with England as a whole[31] and have relatively poor NHS Stop Smoking programme attendance.[13,15,16] We only included community pharmacy chains or independent single pharmacies, which are distinct from those in institutional settings such as NHS hospitals. Consultations had to take place in a private room. Only the adviser and smoker were present though sometimes the consultations were interrupted as they often took place in rooms that doubled as store rooms, which other staff needed to access

We used maximum variation sampling by borough, pharmacy size and length of time on the Stop Smoking programme. Within pharmacies, we invited any staff members certificated through the National Centre for Smoking Cessation and Training (NCSCT) programme, with a mixture of pharmacists and counter assistants therefore included in the study. We explained the study, took consent, and trained them in research methods and governance, so that they could take smoker service user consent and record, securely store and transfer their own consultations to the research team. This meant the natural rhythm of the service was not disrupted. Ethical approval for the study was obtained from the NRES Committee South Central, Berkshire B (reference number 13/SC/0189).

Smokers

All adult smokers recruited onto the NHS Stop Smoking programme during the course of the study were eligible. Smokers were designated as having quit if they self-reported a smoke-free status at 4 weeks after their set quit date and provided an adviser with an expired carbon monoxide (CO) reading of less than 7 parts per million using a CO monitor. Smokers lost to follow up at four weeks were considered still smoking following the Russell criteria.[32]

Data collection

Community pharmacy NHS Stop Smoking programme consultations and prescriptions for medication may continue for up to 12 weeks with formal consultations weekly or two-weekly and informal drop-ins between these times as needed.[7,33] The core period for the programme ends at five to six weeks which tends to equate with the fourth week after the quit date, usually set at the first consultation.[7,33]

Stop Smoking advisers consented eligible smokers and audiorecorded:

1. their first consultation, which tends to set the parameters for the remaining sessions, includes the smoker setting a proximal quit date, and is usually up to three times as long as subsequent sessions (averaging 15 minutes vs 5 minutes);[34]
2. the consultation at 2 weeks post proposed quit date where the adviser explores and attempts to solve any issues in the quit attempt;
3. the consultation four weeks post proposed quit date at which the smoker's quit status is formally designated (as the 4-week quit status) for Public Health England audit statistics and

For peer review only - http://bmjopen.bmj.com/site/about/guidelines.xhtml

pharmacy remuneration[35] (see Sohanpal et al [2016][36] for a discussion of this in relation to our larger study).

We call these weeks 1, 2 and 4 for simplicity. Recordings were anonymised and transcribed as they were received. Pseudo-anonymised metadata (such as demographic data and smoking intensity for the smokers, and training and demographic data for the advisers) were entered with anonymised recordings and transcripts onto a secure clinical trials database.

Matched pairs

We refer to quitters and non-quitters throughout this paper but in consultations 1 and 2 this is a retrospective designation for eventual quitters and non-quitters. We matched 16 pairs of 4-week quitters and non-quitters (i.e. 32 smokers) on gender, ethnicity, age (according to UK Office for National Statistics [ONS] age bands), and smoking intensity (fewer than 10 cigarettes a day, 10-20 a day, more than 20 a day). This was the total number that could be matched on these variables from the data collected. We had initially aimed for 20 matched pairs but obtained saturation at this lower number and so stopped data collection at this point.

Consultations analysis

We used a mixed methods analysis approach, in which themes were identified deductively and inductively and then quantified. Deductive themes were developed from a list of the key linguistic devices used in theme-oriented discourse analysis,[37] which considers how language use constructs professional practice, and the structuring of the medical consultation in the Roter Interaction Analysis System (RIAS),[38] which is a widely used method for coding and quantitatively analysing the conversational strategies used structurally in medical consultations. Our approach thus has its origins in psycholinguistics and its purpose is to look at what talk itself is doing as constitutive of social action, rather than as representational of inner psychological states, behaviours, beliefs or attitudes.[37] To manage the data as we developed the themes, we used a modification of the qualitative content thematic analysis approach outlined in Clark and Braun[39] and Rivas.[40] Thus the core team immersed themselves in the data, reading and re-reading each consultation transcription and meeting to discuss these. CR led development of themes from the data, constantly comparing deductive and emerging inductive themes with each other and also comparing data within themes to ensure each one was discrete and representative of the relevant data. CR operationalised the themes, for a second researcher (RS) to code 20% of the data independently. Differences were reconciled by discussion, until a Cohen's kappa statistic of 76% was obtained.

Once themes were determined, a quantitative summary of these was developed to determine core strategies used by advisers. We compared the frequency of themes between and within successful and unsuccessful quitter consultations, to look for similarities and note any differences. We report differences between the matched pairs in the findings in terms of (eventual) quitters versus non-quitters. We counted the number of times a theme was coded overall and divided this by the number of consultations in which it occurred to get an idea of the intensity within a consultation. We also considered the number of smokers for whom each theme was cited, to get an idea of which themes formed a core set for advisers. This process enabled us to focus on themes that were of high or moderate intensity and core usage and associated with particular outcomes, as well as strategies rarely used but that might be important for service improvement. We then explored these themes in more detail, as reported on here. Given the small numbers involved, tests of significance are not reported here.

For peer review only - http://bmjopen.bmj.com/site/about/guidelines.xhtml

Findings

Participants

Thirty-nine percent (11/28) of recruited advisers provided 158 audiorecordings from 53 smokers. Considering only the 16 matched pairs, these were seen by 9 of the 11 advisers, with a median of three smokers each (range 1-7). Table 1 compares baseline characteristics of the matched pairs to the total sample . Whilst women were under-represented in the matched pairs, other characteristics related to cessation outcome were broadly similar. This includes medication use; though we did not monitor for this systematically, the transcripts indicate that similar numbers of quitters and non-quitters were on vareniciline, for example. Identification (ID) numbers in the tables are also used to attribute extracts from the data in the narrative text. Most advisers in the total group were male (89%) and from pharmacies in City and Hackney (89%). Sixty-seven percent of advisers were Indian, and 11% were from other black and minority ethnic (BME) groups, with missing ethnicity data for one. Importantly, findings were similar whether the adviser was a pharmacist or trained counter assistant. The small number of advisers in the study precluded analysis of the effect of other variables on the consultation. In any case our analysis was focussed on what talk itself does, and was not intended to explore these effects.

VARIABLE	QUITTERS (16)	NON-QUITTERS (16; 6 LTF)	ALL (53; 9 LTF)
FEMALE	3 (19%)	3 (19%)	18 (34%)
AGE (MEDIAN, RANGE)	47 (26-58)	41 (28-59)	41.13 (18-67)
OCCUPATION			
MANAGERIAL AND PROFESSIONAL	2 (13%)	0	5 (9%)
UNEMPLOYED	10 (63%)	10 (63%)	25 (47%)
ROUTINE MANUAL	3 (19%)	3 (19%)	8 (15%)
STUDENT	0	0	1 (2%)
INTERMEDIATE	0	2 (13%)	7 (13%)
RETIRED	0	0	1 (2%)
?NO ANSWER	1 (6%)	4 (25%)	6 (11%)
ETHNICITY			
WHITE BRITISH	11 (69%)	9 (56%)	30 (57%)
SOUTH ASIAN	3 (19%)	3 (19%)	8 (15%)
OTHER WHITE	2 (13%)	3 (19%)	10 (19%)
BLACK AFRICAN	0	0	1 (2%)
BLACK CARIBBEAN	0	0	1 (2%)
MISSING DATA	0	1 (6%)	2 (4%)
CIGARETTES SMOKED			
40	1 (6%)	0	1 (2%)
30	2 (13%)	2 (13%)	5 (9%)
25	3 (19%)	3 (19%)	8 (15%)
20	7 (44%)	9 (56%)	25 (47%)
15	1 (6%)	2 (13%)	5 (9%)
<15	2 (13%)	1 (6%)	9 (17%)

For peer review only - http://bmjopen.bmj.com/site/about/guidelines.xhtml

**PCT**			
**TOWER HAMLETS**	2 (13%)	5	12 (23%)
**CITY AND HACKNEY**	14 (88%)	10	38 (72%)
**NEWHAM**	0	0	3 (6%)

Table 1: Smoker demographic and smoking data summaries for total number sampled and for the matched pair groups (LTF = lost to follow-up).

Themes

Thematic analysis reached saturation, and revealed three overarching themes in the data,

- Negotiating the smoker-adviser relationship
- The roles of the adviser and the smoker in the quit attempt
- Smoker and adviser misalignment on reasons for smoking, relapsing and quitting

These were made up primarily of the subthemes developed deductively from psycholinguistics as described in the methods. Table 2 provides a quantitative overview of the themes, and selected subthemes based primarily on the RIAS and theme-oriented discourse analysis, which we focus on in this paper, and compares quitters and non-quitters. We follow this with a narrative description of themes; extracts are identified by smoker (P), adviser (A) and consultation week (W) while the quantifiers most, common or many, occasionally or some, and a few or rarely, refer to a theme occurrence of 75%+, 50-74%, 25-49% and less than 25%. The first consultation should consider smoker tobacco use and motivation to quit (some advisers used a questionnaire type script) and subsequent ones build on this. Here we focus on what the talk does within any adviser-smoker interaction and the association of interaction strategies per se with quitting or not, hence aggregating the data across consultations, whilst mindful of which consultations they represent. We consider the data by consultation sequence in further analyses to be reported elsewhere, which support the findings of this paper.

From the quantitative data we determined that the core set of strategies used by advisers (occurring in 50% or more of smokers) were: praise; biomedical talk and advice; mention of the side effects of medication; collaborative talk; the use of props and strategies to cope with the urge to smoke; and the use of monitoring and surveillance talk. Significantly, explicit use of motivational talk is not included in this core set.[41] Further strategies were added to the core set in eventual quitters: talk on the importance of support; open door talk; the need for willpower. Managing expectations formed part of the core set for non-quitters only. These themes are discussed in more detail below.

For peer review only - http://bmjopen.bmj.com/site/about/guidelines.xhtml

CODE#	NUMBER OF QUITTERS WITH CODE (% OUT OF n=16)	NUMBER OF NON-QUITTERS WITH CODE (% OUT OF n=16)	NUMBER OF CONSULTATIONS WITH CODE (%)		TOTAL NUMBER OF OCCURRENCES OVERALL	MEAN FREQUENCY OF MENTIONS IN CONSULTATIONS IN WHICH CODE APPEARS
			QUITTERS OUT OF 30 CONSULTATIONS, n (%)	NON-QUITTERS OUT OF 29 CONSULTATIONS n (%)	QUITTERS, NON-QUITTERS	QUITTERS, NON-QUITTERS
**Negotiating the smoker-adviser relationship**						
Lifeworld talk	10 (63%)	9 (56%)	19 (63%)	18 (62%)	75,45	3.95, 2.5
Adviser being non-judgmental	4 (25%)	3 (19%)	6 (20%)	5 (17%)	9, 10	1.50, 2.00
Adviser praise	10 (63%)	9 (56%)	22 (73%)	20 (69%)	64, 53	2.91, 2.65
**The roles of the adviser and the smoker in the quit attempt**						
Receiving biomedical information and advice	14 (88%)	11 (69%)	25 (83%)	16 (55%)	106, 48	4.24, 3.00
Importance of support	8 (50%)	5 (31%)	14 (47%)	6 (21%)	31, 13	2.21, 2.17
Mentioning side effects of medication	9 (56%)	8 (50%)	12 (40%)	11 (38%)	34, 26	2.83, 2.36
Adviser mentioning relapse	4 (25%)	3 (19%)	9 (30%)	6 (21%)	12, 6	1.33, 1.00
Motivational talk	5 (31%)	5 (31%)	15 (50%)	12 (41%)	37, 34	2.47, 2.83
Confidence in being able to quit	3 (19%)	1 (6%)	8 (27%)	2 (7%)	8, 3	1.00, 1.50
Need for willpower	8 (50%)	7 (44%)	9 (30%)	13 (45%)	17, 18	1.89, 1.38
Collaborative talk (e.g. ‘we’)	10 (63%)	8 (50%)	20 (67%)	15 (52%)	67, 51	3.35, 3.40
Ownership of the quit	5 (31%)	4 (24%)	8 (27%)	5 (17%)	16, 6	2.00, 1.20
Managing smoker expectations of the programme	5 (31%)	9 (56%)	9 (30%)	11 (38%)	19, 30	2.11, 2.73

For peer review only

For peer review only - http://bmjopen.bmj.com/site/about/guidelines.xhtml

The use of quit props and strategies	11 (69%)	10 (63%)	22 (73%)	22 (76%)	73, 39	3.32, 1.77
'Open door' talk (invitations to informal consultations between the formal ones)	9 (56%)	7 (44%)	10 (33%)	8 (28%)	15, 12	1.50, 1.50
Monitoring and surveillance	14 (88%)	15 (94%)	21 (70%)	17 (59%)	38, 38	1.81, 2.24
**Smoker and adviser misalignment on reasons for smoking, relapsing and quitting**						
Smoker explanations for smoking						
Stress	6 (38%)	9 (56%)	8 (27%)	13 (45%)	19, 17	2.38, 1.31
Social factors	4 (25%)	3 (19%)	6 (20%)	4 (14%)	8, 6	1.33, 1.50
Financial reasons	5 (31%)	5 (31%)	5 (17%)	6 (21%)	6, 8	1.20, 1.33
Health problem	8 (50%)	11 (69%)	8 (27%)	13 (45%)	19, 32	2.38, 2.46
Smoker explanations for wanting to quit						
Quitting for families (including children)	5 (31%)	3 (19%)	6 (20%)	3 (10%)	15, 3	2.50, 1.00
Obligations to self and others	2 (13%)	1 (6%)	4 (13%)	1 (3%)	6, 1	1.50, 1.00
Negative feelings towards smoking	1 (6%)	3 (19%)	1 (3%)	5 (17%)	1, 5	1.00, 1.00
Negative impact of smoking on appearance (e.g. Ageing effects) or identity (e.g. Smelly)	1 (6%)	3 (19%)	2 (7%)	5 (17%)	2, 8	1.00, 1.60
Adviser-suggested motivator for smoker to quit						
Financial	4 (25%)	6 (38%)	10 (33%)	8 (28$)	14, 9	1.40, 1.13
Health scare tactics	4 (25%)	3 (19%)	5 (17%)	3 (10%)	8, 5	1.60, 1.67

# Themes shown here have been selected from a larger pool for this paper.

For peer review only - http://bmjopen.bmj.com/site/about/guidelines.xhtml

Table 2: Quantitative comparisons between quitters and non-quitters in: a) number of quitters and non-quitters for which each code was identified (second and third columns); b) number of consultations overall in which each code was identified (middle columns); c) total number of occurrences in all consultations (penultimate column); d) relative frequency with which codes were used in individual consultations (final column).

1. Negotiating the smoker-adviser relationship

Lifeworld talk was common across the groups, though occurring in slightly more eventual quitters and with more occurrences within their consultations. This is talk about a person's experience of life with all its contextual and situational nuances.[42-45] Through lifeworld talk, the advisers gained a shared understanding with the smoker of the context of the smoker's quit attempt and the individual difficulties they faced. The adviser could then discuss approaches to quitting that took into account such things as the stress that the smoker in the following extract describes.

.

> A: So whilst you're on Champix and you left work and you're a bit stressed did you say?
> S: Yeah, because there's many changes in the work you remember when you told me in the beginning, like try to, don't change the work and the things. But they change our store manager, my manager is leaving. The work we used to do, six persons now going to be in two persons, oh my god. It's just,
> A: So you're a bit more stressed at work.
> S: Terrible. It's terrible.
> A: So you came home and you're a bit stressed still, so you felt like you wanted a cigarette? But you didn't have a cigarette? (P807-A81-W4, quit)

The advisers made it clear to around one quarter of smokers that they were not going to judge them for any lapses and failures, as a way to motivate them to continue the programme, illustrated in the following extract. This is in line with good practice.

> A: Fantastic. So again, my idea is to mentor you and support you and that's what it's about, yeah? I'm not here to tell you off. It's about where you're finding it hard and what we need to work together to find a solution for you. (P107-A1-W1, quit)

Praise was common across groups, but with marked qualitative differences. Quitters received limited and brief praise. By contrast, eventual non-quitters were given extensive (extreme formulation[46,47]) praise even when they had not achieved the NCSCT-recommended 'not a puff', as below.

> S: Well, it's actually helped a lot, so I'm only smoking around ten.
> A: Ten plus? Ten to fifteen?
> S: Ten plus.
> A: That's fantastic. In the space of six, seven days you've cut down by over half. (P4-A1-W2, not quit)

2. The roles of the adviser and the smoker in the quit attempt

Biomedical information and advice was imparted across the groups but quitter consultations were most heavy with it. This may mean they were more open to biomedical talk or that the adviser got an impression that they were, or it may be an incidental finding. Side effects of the medication and withdrawal symptoms from stopping smoking, including weight gain, were discussed with many smokers. The possibility of relapse was discussed with only 25% of quitters and 19% of non-quitters.

For peer review only - http://bmjopen.bmj.com/site/about/guidelines.xhtml

There was some compensation in the degree to which the use of strategies to deal with cravings (hence *risk* of relapse) was commonly encouraged (see extract later in this section).

Advisers made attempts to manage many of the eventual non-quitters' expectations of the programme as well as emphasising their required commitment. Both strategies were undertaken only occasionally with quitters. Many quitters but only some non-quitters were told of the importance of advisers' counselling support. Advisers' motivational talk was occasional in both groups, and did not form part of the core set of strategies and advisers only rarely asked for (and therefore got) expressions of confidence in being able to quit (3 quitters over 8 consultations vs 1 non-quitter over 2 consultations), a specific element of motivational interviewing. The need for the smoker to draw on their own willpower to quit was considered in most quitters but only some non-quitters, and the necessary balance between adviser support and more personal willpower or motivation was only discussed with one non-quitter (in the following extract) and one quitter.

> S: I've got a photograph of me taken when I was in intensive care in hospital, I should take a look at that every time I want a cigarette.
> A: Perfect! You need some motivation.
> S: I don't want to end up like that again.
> A: Exactly, you need that motivation. And if there's anyone else that can help you also...
> S: My mum, yeah, she's very supportive.
> A: Sure. You need that support as well. But you've got us as well because you'll be coming here every week. (P806-A81-W1, not quit)

Collaborative talk (such as the use of the phrase "we will succeed"), a supportive stratagem, was common in both groups. The related topic 'smoker's ownership of the quit' was rarely mentioned in non-quitter consultations and only slightly more so in quitters'. Some messages were implied rather than explicit. The use of props and strategies to deal with cravings requires some ownership and self-management of the quit and was recommended at least once in a majority of smokers and consultations, though more frequently discussed *within* quitter consultations. Many different props and strategies were suggested, with the following only one example.

> A: I want you to make sure you start off in the morning; you've got your bottle of 1.5 litres to 2 litres. Your aim, you start in the morning, you finish at the end of the day. Every time you want a cigarette, you're going to drink the water. You're going to complete it. (P101-A11-W1, not quit).

The advisers invited many eventual non-quitters to drop into the pharmacy for support and advice any time the shop was open, a particular advantage that pharmacies have over other healthcare settings. Such 'open door' talk, offering a more informal variant of adviser support, was said to fewer eventual non-quitters and was less successful for these.

In most smokers, the CO monitor was depicted by the adviser as a monitoring or surveillance tool that was used to check the smoker's progress and even catch the smoker out:

> A: Well, it's good that you're here, we will help you in any way we can. What I ask you every week is if I can take a reading from you. This is called a CO monitor and this machine measures the amount of carbon monoxide inside of your system. And I call this my lie detector. I'm not going to say that you're going to lie to me, but if you do come in and I say have you had any cigarettes, and you say no, I haven't had any, this will tell me the truth. (P7-A1-W1, quit)

For peer review only - http://bmjopen.bmj.com/site/about/guidelines.xhtml

3. Smoker and adviser misalignment on reasons for smoking, relapsing and quitting

An actual health problem or worry about getting one was cited as reason to quit by more than half the smokers and was invoked slightly more commonly by non-quitters. There was an inverse relationship between this and adviser use of health scare tactics to motivate the smoker, though these tactics were relatively uncommon. Financial reasons for wanting to quit were cited by just under one third of smokers. However the advisers tried to motivate the smokers with talk of financial savings on 23 separate occasions. Misalignment is demonstrated in the following extract for example.

> S: I mean I don't want to die. I don't want to have a stroke.
> A: So that's your main impulse now and more likely you'll succeed because of that.
> S: I don't want to end up like that…
> A: The other reasons why maybe you feel I'll give up now, maybe save a bit of money or whatever before…
> S: That's the backburner, that's nothing to do…
> A: Exactly. Was that the reasons before?
> S: No.
> A: What were the reasons before that you wanted to stop smoking?
> S: Smell; I don't like the smell. I've got this thing, when I eat, people smoke around me, I don't like it, I don't like the smell of it when I'm eating. People light one up when you're having something to eat and oh, it's disgusting, I don't like the smell. (P806-A81-W1, not quit)

Quitters were twice as likely to mention their families (including children) as motivation though under a third did so. Other reasons for smoking were cited relatively infrequently. Notably the misalignment or mismatch between smoker declaration of health as motivation to quit and adviser comments on health problems caused by smoking was more likely in non-quitters, and advisers were also less likely to mention motivational factors at all to eventual non-quitters. In quitters' consultations, advisers were likely to mention motivators, especially financial, even when the smoker did not.

Discussion

We applied rigorous and systematic analytical methods to a sample of audiorecordings of real world community pharmacy adviser-smoker NHS Stop Smoking consultations with the premise that the community pharmacy Stop Smoking programme should be person-centred. Specifically, we explored associations of thematic patterns in the verbal interactions with 4-week quit success. Although there are a number of ethnographic studies of client-pharmacy staff communication processes or interactions[24,38,48-71] there is none on stopping smoking in the community. Our overall findings accord with those of Pilnick's study[49-51] in a UK hospital paediatric outpatient clinic pharmacy, the most comparable study to ours, which focussed on advice-giving in the counselling role, but we provide rich new data.

There were some clinically potentially significant omissions across the data. For example talk about relapse was rare, though there was some compensation in the degree to which the use of strategies to deal with cravings was frequently encouraged and the biomedical problems of quitting often explored. We found many examples of good practice, including significant evidence of lifeworld talk,[42,43] which was common across the two groups. Mishler,[43] applying the Habermas theory of Communicative Action[72] to medical encounters, itself derived from Husserl's phenomenological concept of consciousness and subjectivity,[44] showed how this enables person-centred support. It provides the pharmacy advisers with the smokers' own nuanced and contextualised[73-81] accounts of

For peer review only - http://bmjopen.bmj.com/site/about/guidelines.xhtml

their personal daily lives, in which they manage their quit attempts. When the adviser uses these to shape their recommendations, advice and medical support, adviser consultations are more likely to be effective.[44,82] The occurrence of lifeworld talk in our data suggests that the community setting and the advisers' social familiarity[81] with the smokers as customers enables such smoker-centred care, in a way that is potentially greater than is possible in GP consultations. This supports other research suggesting that community pharmacies are ideally placed for smoking cessation and other health behaviour change tasks.[10-13]

Nonetheless, the core set of strategies common across both quitter and non-quitter groups focussed on the practical and the medical, biomedical talk and advice (the 'voice of medicine'[42,43]). This was certainly an important part of consultations, and appropriate given the pharmacy staff member's role as *adviser* and the need for such talk in the first consultation in particular. But unlike biomedical talk, lifeworld talk was not included in this core set, in other words it was less consistently used than. We found, from more detailed analysis, that differences between smokers in the way lifeworld talk was used could be related to quit success or failure.

It appeared that advisers used lifeworld talk to make judgments about likely smoker quit success. This then compromised its effective use. We found this affected non-quitters disproportionately, and this seemed due to the adviser focussing on possible problems in those they judged as less likely to quit, and making assumptions about reasons the smoker found it hard to quit. This counselling approach was not used in those who went on to quit. Our data show that in this way, pre-judgements led to three sources of misalignment with, or confusion in, the smokers who did not quit. First, advisers tended to offer casual 'open door' drop-in support between consultations more often to eventual non-quitters, perhaps because they perceived they needed more support. Bu these offers were not clear or explicit in their purpose and appear to have simply confused smokers as to when they were meant to attend. Second, praise and willpower talk were common in both quitters and non-quitters but the way they were communicated was often poorly matched to smoker effort. Extreme statements made in praise of non-quitters when they were still smoking created cognitive dissonance. Third, the advisers drew on financial and health benefits as motivators for quitting in those who then failed to quit and had cited other reasons for wanting to quit. The misalignment between the smoker's reasons for smoking or wanting to quit, and the adviser's perceptions of what these were, would result in the adviser setting inappropriate goals for the smoker.[72-82]

These misalignments may therefore explain why a smoker's sharing of their lifeworld was not always associated with quit success. This interpretation of the data is supported by similar findings in our separate analysis of adviser interviews, with some advisers explicitly stating that they emphasised particular strategies in those smokers they considered would find it harder to quit, to try to help them.[36] Misalignments push interactants 'out of sync' with each other, making it hard for them to achieve a common goal. Nyugen considers how misalignments in pharmacy counselling need to be delicately managed in order not to affect the tenor of the rest of the conversation.[62] Misalignments were noted by Pilnick[49-51] as problematizing the adviser's advice giving but we have shown how they develop to create issues in the adviser's cognitive development of what that advice might be as well as how their counselling support should be configured.

Our data also show that the advisers drew on sometimes apparently contradictory strategies to induce a quit. For example, they often combined reassurance of a non-judgmental approach with the use of the CO monitor as a surveillance tool. While this seems to have been successful, biomedical talk and advice, talk of being non-judgmental, monitoring and surveillance, and the advisers' use of authority and expertise to prejudge likely smoker success, are all premised on a

For peer review only - http://bmjopen.bmj.com/site/about/guidelines.xhtml

power differential between adviser and smoker and may therefore appear to sit in dialectical tension with the development of shared understandings and social familiarity from the community setting. Social familiarity and power differentials are not mutually exclusive but they are less likely to co-exist, the greater the difference in social status between two interactants.[72,78,81,83,84] Thus the pharmacy community setting provides particular communication issues that are less likely to occur in encounters with doctors and medical specialists. Social familiarity is shown in our data to have the potential to enhance quit success and power differentials to compromise this, and the best outcomes might be achieved when advisers strike a balance between the two, optimising the benefits of the community pharmacy setting. Figure 1 demonstrates this. Considering recommendations for pharmacy adviser smoking cessation care, we found successful quits to be more likely when there was more smoker-centred care with counselling plans that were based on the smokers' own lifeworlds, and that matched strategies such as praise to the smokers' actual rather than predicted behaviours.

Strengths and limitations

Our study contributes to current knowledge and practice with novel data from a pharmacy service that has not previously been subject to ethnographic study. As this was a qualitative study we considered data from a small and varied sample of participating staff members, smokers and community pharmacies, adding to richness of the findings. Since we were comparing two groups, we matched them on relevant variables to increase the dependability of our analysis. We achieved theme saturation within this matched pair design, with adequate agreement between coders, group data sessions and consideration of negative cases in the data adding to credibility and dependability of findings. Themes reported in this analysis correspond with some of the themes developed separately from semi-structured qualitative interviews with advisers and smokers from the same study.[36] Our study has enabled us to identify key elements of talk and can be used to strengthen consultations and make them more effective.

Our study also has limitations. We were not successful in recruiting pharmacy chains and make no claims to transferability of the findings to other pharmacies, particularly those in more affluent areas. Further, it may not be possible to extrapolate findings from these consultations, which were undertaken in a private room, to over the counter community pharmacy interactions. In collecting naturalistic qualitative data, our aim was rather to provide in depth exploration of communication practices. This was usefully achieved. A number of contextual and demographic variables are known to affect pharmacist-smoker communication and quit successes.[85] We were unable to explore these in our small sample. We did not case match by adviser ethnicity or other adviser variables as this would have required oversampling for our specific research question, which was not intended to explore this, but this might be a topic for different research. However we systematically looked for adviser-specific patterns and found that our analysis held irrespective of adviser. Participants were not blinded to the nature of the research and had a potential vested interest in successful quits, creating a possible performance effect. However the unobtrusive data collection method will have reduced this risk with smokers and the effect would have been the same for quitters and non-quitters. There may have been an intrinsic difference between the quitters and non-quitters before they even entered the consultation room and our study was not designed to explore this. For example, although baseline characteristics known to be related to quitting, such as gender and age were taken into account we did not systematically collect data on other potentially significant variables such as number of previous quit attempts or medication use. Our thematic analysis suggests this would have been useful. The quitters may have been successful irrespective of consultation features; the availability of support itself may be sufficient.[41,85]

For peer review only - http://bmjopen.bmj.com/site/about/guidelines.xhtml

Clinical implications

Community pharmacy Stop Smoking advisers may need to reflect on the inconsistencies in their delivery of and the content of the consultations. They should begin with consideration of their use of the considerable lifeworld talk generated by smokers through their social familiarity with the pharmacy advisers. Lifeworld talk should be used to modify adviser counselling to suit key external psychosocial factors, namely the *context* within which the quit attempt is made and the related *motivators* to quitting. Our data suggest that currently advisers may instead modify the consultation to suit their assumptions about internal psychological factors such as the sm*oker's capacity to quit or motivation for quitting*.[41] In this way advisers sometimes develop the consultation in ways that, while well-meaningly intended, run the risk of being counter-productive. For smokers judged by them as less likely to quit this may include inappropriately overemphasising some support strategies (such as praise, managing expectations) and underplaying others (such as the need for willpower and the importance of adviser support). Our data illustrate the potential for misalignment that this creates. Moreover, the exaggerated praise we saw in non-quitter reductions in smoking conflicts with the 'not a puff' rule recommended by the NCSCT. Advisers should use findings from our study to clarify where they might usefully capitalise on the social closeness they seem to share with the smokers they see, with more appropriate use of lifeworld talk and more use of smoker-declared motivators during motivational interviewing.

Recommendations for research

Our study only considers short-term outcomes, something we are addressing with further research. The analyses have been used to inform the development and content of our training intervention for pharmacy Stop Smoking advisers, evaluated in a cluster randomised trial (grant number RP-PG-0609-10181). We are currently exploring the micro-detail of the talk in the consultations using conversation analysis with a view to further dissemination and use. Our study could be replicated in less disadvantaged areas or in specific minority groups.

4487 words

Acknowledgments The authors thank participants and their community pharmacies. This paper presents independent research funded by the National Institute for Health Research (NIHR) under its Programme Grants for Applied Research Programme. The views expressed are those of the authors and not necessarily those of the NHS, the NIHR or the Department of Health.

Contributors RW conceived the study. CR undertook the analysis and wrote the manuscript. RS, LA and VM were involved in data collection. RS, LS and RW contributed to the data analysis and interpretation. VM, LS, LA, EE, CG, ST, SE and RW were involved in revising the article critically and contributing important intellectual content. RW is the guarantor.

Competing interests None.

Funding: NIHR Programme grant RP-PG-0609-10181

References

For peer review only - http://bmjopen.bmj.com/site/about/guidelines.xhtml

References

1. Gidman W, Ward P, McGregor L. Understanding public trust in services provided by community pharmacists relative to those provided by general practitioners: a qualitative study. *BMJ Open* 2012;2:3 e000939 doi:10.1136/bmjopen-2012-000939
2. The Royal Pharmaceutical Society of Great Britain. *Pharmacy in a New Age: The New Horizon.* London: The Royal Pharmaceutical Society of Great Britain, 2006.
3. Department of Health. *Pharmacy in England: Building on strengths – delivering the future.* London: The Stationery Office, 2008.
4. Department of Health. *Speech by the Rt Hon Alan Johnson MP, Secretary of State for Health NHS Confederation Primary Care Network,* 2008 webarchive.nationalarchives.gov.uk/+/www.dh.gov.uk/en/MediaCentre/Speeches/Speecheslist/DH_083369 (accessed 21 December 2015)
5. Royal Pharmaceutical Society. *The Changing Face of Pharmacy,* https://www.rpharms.com/public-affairs-pdfs/rps-changing-face-of-pharmacy-booklet.pdf (accessed 9 January 2016)
6. NHS England. *Improving health and patient care through community pharmacy - a call to action,* 2013. http://www.england.nhs.uk/wp-content/uploads/2013/12/community-pharmacycta.pdf (accessed 24 Feb 2015)
7. NHS Employers. *Pharmacy-based stop smoking services: optimising commissioning*. London: NHS Employers, 2009.
8. Pharmaceutical Services Negotiating Committee, *The community pharmacy: A guide for general practitioners and practice staff.* July 2013.
9. NHS Confederation. *Health on the high street. Rethinking the role of community pharmacy,* London, 2013. www.nhsconfed.org/Publications/reports/Pages/Health-on-high-street-rethinking-community-pharmacy.aspx (accessed 24 Feb 2015)
10. National Pharmacy Association. *Pharmacy Health Trainers Resource*, 2009.
11. Fajemisin F. *Community Pharmacy and Public Health. Solutions for Public Health*, 2013. URL: http://www.sph.nhs.uk/sph-documents/community-pharmacy-and-public-health-finalreport/?searchterm=community pharmacy (accessed 21 Dec 2015)
12. Hametz I, Hodgkiss F, Sheehy C. *Review of the community pharmacy public health service for smoking cessation and emergency hormonal contraception*. Scottish Government, Edinburgh; 2011
13. Todd A, Copeland A, Husband A, Kasim A, Barbra C. The positive pharmacy care law: an area-level analysis of the relationship between community pharmacy distribution, urbanity and social deprivation in England, *BMJ Open* 2014;4:8 e005764 doi:10.1136/bmjopen-2014-005764
14. Ali A, Wright N, Rae M. *Addressing Health Inequalities – A guide for general practitioners*. Royal College of General Practitioners Health Inequalities Standing Group. London: RCGP, 2008
15. West R, May S, West M, Croghan E, McEwen A. Performance of English stop smoking services in first 10 years: analysis of service monitoring data. *BMJ* 2013;47:f4921.
16. Anon. Community pharmacy: At the heart of public health. Pharmaceutical Services Negotiating Committee; 2013
17. Lifestyle statistics, Health and Social Care Information Centre. *Statistics on NHS Stop Smoking Services: England, April 2015 – March 2016,* 2016
18. Bauld L, Hiscock R, Dobbie F, Aveyard P, Coleman T, Leonardi-Bee J, McEwen, A. English Stop-Smoking Services: One-Year Outcomes. *International Journal of Environmental Research and Public Health 2016;*13(12):1175. http://doi.org/10.3390/ijerph13121175

For peer review only - http://bmjopen.bmj.com/site/about/guidelines.xhtml

19. Bartlett EE, Grayson M, Barker R, Levine DM, Golden A, Libber S. The effects of physician communications skills on patient satisfaction; recall, and adherence. *J Chronic Dis.* 1984;37(9–10):755–764
20. Epstein RM, Street RL. The Values and Value of Patient-Centered Care. *Annals of Family Medicine* 2011;9(2):100-103. doi:10.1370/afm.1239
21. The Health Foundation. *Person-centred care made simple: What everyone should know about person-centred care*, The Health Foundation, London 2014.
22. Department of Health. Equity and Excellence: Liberating the NHS. Department of Health: London, July 2010
23. National Institute for Health and Care Excellence. *Falls in older people: assessing risk and prevention. NICE guidelines [CG161]*, June 2013
24. Royal Pharmaceutical Society Wales. *Your Care, Your Medicines: Pharmacy at the heart of patient-centred care*, 2015
25. Greenhill N, Anderson C, Avery A, Pilnick A. Analysis of pharmacist-patient communication using the Calgary-Cambridge guide. *Patient Education and Counseling* 2001; (83): 423-431
26. Salter C, Holland R, Harvey I, Henwood K. 'I haven't even phoned my doctor yet'. The advice giving role of the pharmacist during consultations for medication review with patients aged 80 or more: qualitative disclosure analysis. *BMJ* 2007; May 26;334(7603):1101
27. Stahlke Wall S. Focused Ethnography: A methodological adaptation for social research in emerging contexts. *Forum Qualitative Sozialforschung / Forum: Qualitative Social Research*, [S.l.], v. 16, n. 1, nov. 2014. ISSN 1438-5627. Available at: <http://www.qualitative-research.net/index.php/fqs/article/view/2182>. Date accessed: 14 may 2017.
28. Knoblauch H. Focused ethnography. *Forum Qualitative Sozialforschung / Forum: Qualitative Social Research, [S.l.]* 2005; 6(3), ISSN 1438-5627. Available at: <http://www.qualitative-research.net/index.php/fqs/article/view/20>. Date accessed: 29 apr. 2017.
29. Steed L, Sohanpal R, Yee W, Rivas C, Jumbe S, Chater A, Todd A, Edwards E, MacNeill V, Macfarlane F, Greenhalgh T, Griffiths C, Eldridge S, Taylor S, Walton R. Equipping community pharmacy workers as agents for health behaviour change: developing and testing a theory-based intervention. *BMJ Open* (accepted)
30. Wolf ZR Ethnography: the method. In Munhall PL (Ed) *Nursing Research – A Qualitative Perspective.* Fourth edition. 2005, Jones and Bartlett, Sudbury MA
31. Department for Communities and Local Government, *English indices of deprivation 2015*, Department for Communities and Local Government, London, 2015
32. West R, Hajek P, Stead L, Stapleton J. Outcome criteria in smoking cessation trials: proposal for a common standard. *Addiction* 2005;100: 299–303. doi: 10.1111/j.1360-0443.2004.00995.x
33. National Institute for Health and Care Excellence. *Training in smoking prevention and cessation.* NICE, 2015.
34. Bauld L, Chesterman J, Ferguson J, Judge K. A comparison of the effectiveness of group-based and pharmacy-led smoking cessation treatment in Glasgow. *Addiction* 2009; 104: 308-16
35. Bauld L, Debbie F. *Local Stop Smoking Services Service and delivery guidance 2014*, 2014 National Centre for Smoking Cessation and Training
36. Sohanpal R, Rivas C, Steed L., MacNeill V, Kuan V, Edwards E, Griffiths C, Eldridge S, Taylor S, Walton R. Understanding recruitment and retention in the NHS community pharmacy stop smoking service: perceptions of smoking cessation advisers. *BMJ Open* (submitted).
37. Roberts C, Sarangi S. Theme-oriented discourse analysis of medical encounters. *Medical Education* 2005;39,632–640. doi:10.1111/j.1365-2929.2005.02171.x

For peer review only - http://bmjopen.bmj.com/site/about/guidelines.xhtml

38. Cavacoa A, Roter D. Pharmaceutical consultations in community pharmacies: utility of the Roter Interaction Analysis System to study pharmacist–patient communication. *IJPP* 2010, 18: 141–148
39. Braun V, Clarke V. *Thematic analysis.* In: Cooper, H., ed. (2012) *APA Handbook of Research Methods in Psychology.* (Vol. 2) Washington, DC: American Psychological Association).
40. Rivas C. *Coding qualitative data*. In, Seale, Clive (ed.) *Researching Society and Culture. Third Edition.* London, GB, Sage
41. Rollnick S, Miller WR. What is Motivational Interviewing? *Behavioural and Cognitive Psychotherapy* 1995;23:325-334
42. Hemingway A. Lifeworld-led care: is it relevant for well-being and the fifth wave of public health action? *Int J Qual Studies on Health and Well-being* 2011;6 (4), Article ID 10364
43. Mischler EG. *The discourse of medicine. The dialectics of medical interview*s, Ablex, Norwood, NJ, 1984
44. Husserl E. *The crisis of European science and transcendental phenomenolog*y, Northwestern University Press, Evanston, Ill, USA, 1970
45. Barry CA, Stevenson FA, Britten N, Barber N, Bradley CP. Giving a voice to the lifeworld. More humane, more effective medical care? A qualitative study of doctor-patient communication in general practice. *Soc Sci Med* 2001; 53, 487–505
46. Edwards D. Extreme case formulations: softeners, investment, and doing nonliteral. *Research on Language and Social Interaction* 2000;33(4), 347–373
47. Pomerantz A. Extreme case formulations: A way of legitimizing claims. *Human Studies* 1986; 9*,* 219–229
48. Shah B, Chewning B. Conceptualizing and measuring pharmacist-patient communication: a review of published studies. *Res Soc Admin Pharm* 2006; 2: 153–185
49. Pilnick A. The interactional organization of pharmacist consultations in a hospital setting: a putative structure. *J Pragmatics* 2001; 33, 1927–1945
50. Pilnick A. "Patient counseling" by pharmacists: advice, information, or instruction? *Sociol Quart* 1999; 40, 613–622
51. Pilnick A. "Patient counselling" by pharmacists: four approaches to the delivery of counselling sequences and their interactional reception. *Soc Sci Med* 2003; 56, 835–849.
52. Garner M, Watson MC. Using linguistic analysis to explore medicine counter assistants' communication during consultations for nonprescription medicines. *Patient Educ Couns* 2007; 65,51–57
53. Stevenson FA. Achieving visibility? Use of non-verbal communication in interactions between patients and pharmacists who do not share a common language. *Sociology of Health & Illnes*s 2014; 36(5), 1467. DOI:10.1111
54. Stevenson FA, Leontowitsch M, Duggan C. Over-the-counter medicines: professional expertise and consumer discourses. *Sociol Health Illn* 2008;30,913–928
55. Leontowitsch M, Stevenson F, Nazareth I, Duggan C.'At the moment it is just a couple of eccentrics doing it': concordance in day-to-day practice. *Int J Pharm Pract* 2005; 13, 273–280
56. Evans SW, John DN. A preliminary investigation of the interactions between UK and US community pharmacists and their prescription clients. *Int J of Pharmacy Practice* 1995; 3: 157–162. doi: 10.1111/j.2042-7174.1995.tb00810.x
57. Pelicano-Romano J, Neves MR, Amado A, Cavaco AM. Do community pharmacists actively engage elderly patients in the dialogue? Results from pharmaceutical care consultations. *Health Expectations* 2015, 18: 1721–1734. doi: 10.1111/hex.12165
58. Chen J, Britten N. 'Strong medicine': an analysis of pharmacist consultations in primary care. *Fam Pract* 2000;17, 480–483

For peer review only - http://bmjopen.bmj.com/site/about/guidelines.xhtml

59. Babalola O, Erhun WO. Pharmacist medication history interview of elderly patients in a teaching hospital in Nigeria. *Int J Pharm Pract* 2001;9, 97–103
60. Salter C. Compliance and concordance during domiciliary medication review involving pharmacists and older people. Sociol Health Illn, 32 (2010), pp. 21–36
61. Hargie OD, Morrow NC, Woodman C. Pharmacists' evaluation of key communication skills in practice. *Patient Educ Couns* 2000;39, 61–70
62. Dyck A, Deschamps M, Taylor J. Pharmacists' discussions of medication side effects: a descriptive study. *Patient Educ Couns* 2005; 56, 21–27
63. Nguyen HT. Boundary and alignment in multiparty conflict talk. *J Pragmatics* 2011; 43, 1755–1771
64. Bissell P, Ward P, Noyce PR. Appropriateness measurement: application to advice-giving in community pharmacies. *Soc Sci Med* 2000;51, 343–359
65. Skoglund P, Isacson D, Kjellgren DI. Analgesic medication–communication at pharmacies. *Patient Educ Couns* 2003; 51,155–161
66. Montgomery AT, Kettis Lindblad A, Eddby P, Soderlund E, Tully MP, Kalvemark Sporrong S. Counselling behaviour and content in a pharmaceutical care service in Swedish community pharmacies. *Pharm World Sci* 2010;32,455–463
67. Watermeyer J,Penn C. "Tell me so I know you understand": pharmacists' verification of patients' comprehension of antiretroviral dosage instructions in a cross-cultural context. *Patient Educ Couns* 2009;75, 205–213
68. Watermeyer J. "Now here come the pills that are going to save your life": pharmacists' discussions of antiretroviral drugs in a context of life and death. *AIDS Care* 2011; 23,807–813
69. Watermeyer J, Penn C. The organization of pharmacist-patient interactions in an HIV/aids clinic. *J Pragmatics* 2009, 41,2053–2071
70. Ylanne V, John DN. Roles of medicines counter assistants in advice giving in community pharmacies: a discourse analysis. *Pharm World Sci* 2008;30, 199–2077
71. Paluck EC, Green LW, Frankish CJ, Fielding DW, Haverkamp B. Assessment of communication barriers in community pharmacies. *Eval Health Prof* 2003 26: 380
72. Habermas J. *The theory of communicative action, Reason and the rationalization of society,* vol. 1. London: Heinemann, 1984
73. Seale C, Rivas C, Kelly M. The challenge of communication in interpreted consultations in diabetes care: a mixed methods study. *The British Journal of General Practice* 2013;63(607):e125-e133. doi:10.3399/bjgp13X663082.
74. Webb H. 'I've put weight on cos I've bin inactive, cos I've 'ad me knee done': moral work in the obesity clinic. *Sociology of Health and Illness* 2009; 31 (6), 854–871
75. Heritage J, Lindstrom A. Motherhood, medicine, and morality: scenes from a medical encounter. *Research on Language and Social Interaction* 1998; 31, 397–438
76. Heritage J, Robinson JD. *Accounting for the visit: giving reasons for seeking medical care*. In: J. Heritage, D.W. Maynard (Eds.), Communication in medical care: Interaction between primary care physicians and patients, Cambridge University Press, Cambridge, 2006, pp. 48–85
77. McMullen LM, Herman J. Women's accounts of their decision to quit taking antidepressants. *Qualitative Health Research* 2009;19 (11), 1569–15
78. Peräkylä A. Authority and accountability: the delivery of diagnosis in primary health care. *Social Psychology Quarterly* 1998;61 (4), 301–320
79. Roberts C, Sarangi S, Moss B. Presentation of self and symptoms in primary care consultations involving patients from non-English speaking backgrounds. *Communication & Medicine* 2004; 1 (2),159–169
80. Stephens C, Breheny M. Menopause and the virtuous woman: the importance of the moral order in accounting for medical decision making. *Health* 2008; 12, 7–24

For peer review only - http://bmjopen.bmj.com/site/about/guidelines.xhtml

81. Seale C, Rivas C, Al-Sarraj H, Webb S, Kelly M. Moral mediation in interpreted health care consultations, *Social Science & Medicine* 2013; 98, 141-148, ISSN 0277-9536, http://dx.doi.org/10.1016/j.socscimed.2013.09.014.
82. Britten N, Stevenson FA, Barry CA, Barber N, Bradley CP. Misunderstandings in prescribing decisions in general practice: qualitative study *BMJ* 2000; 320 :484.
83. Trinkaus Zagzebski L. Epistemic authority: a theory of trust, authority, and autonomy in belief, Oxford University Press, Oxford, 2012
84. Goffman E. *Interaction Ritual: Essays on face-to-face behavior.* (New York: Doubleday Anchor, 1967) (London: Allen Lane, 1972)
85. Lancaster T, Stead LF. Individual behavioural counselling for smoking cessation. *Cochrane Database of Systematic Reviews* 2005, Issue 2. Art. No.: CD001292. DOI: 10.1002/14651858.CD001292.pub2.

Figure 1: Tentative model of misalignment in the community pharmacy NHS Stop Smoking service consultation.

Funding: NIHR Programme Grants for Applied Research Programme (RP-PG-0609-10181). Disclaimer This article presents independent research funded by the National Institute for Health Research (NIHR) under its Programme Grants of Applied Research Programme. The views expressed are those of the authors and not necessarily those of the NHS, the NIHR or the Department of Health.

Data sharing statement. This study analyses qualitative data and the advisers and smokers did not consent to have their full consultation transcripts made publicly available. They consented for their data to be stored for 5 years, as part of the 5-year programme grant project, and to be used in this or other studies directly linked to the 5-year project. Supporting excerpts from the raw data (quotes from consultations) are available within the text of the paper. Full transcripts (with identifying information removed) will be available, following completion of the project, on request from the study guarantor, Robert Walton, r.walton@qmul.ac.uk

For peer review only

For peer review only - http://bmjopen.bmj.com/site/about/guidelines.xhtml

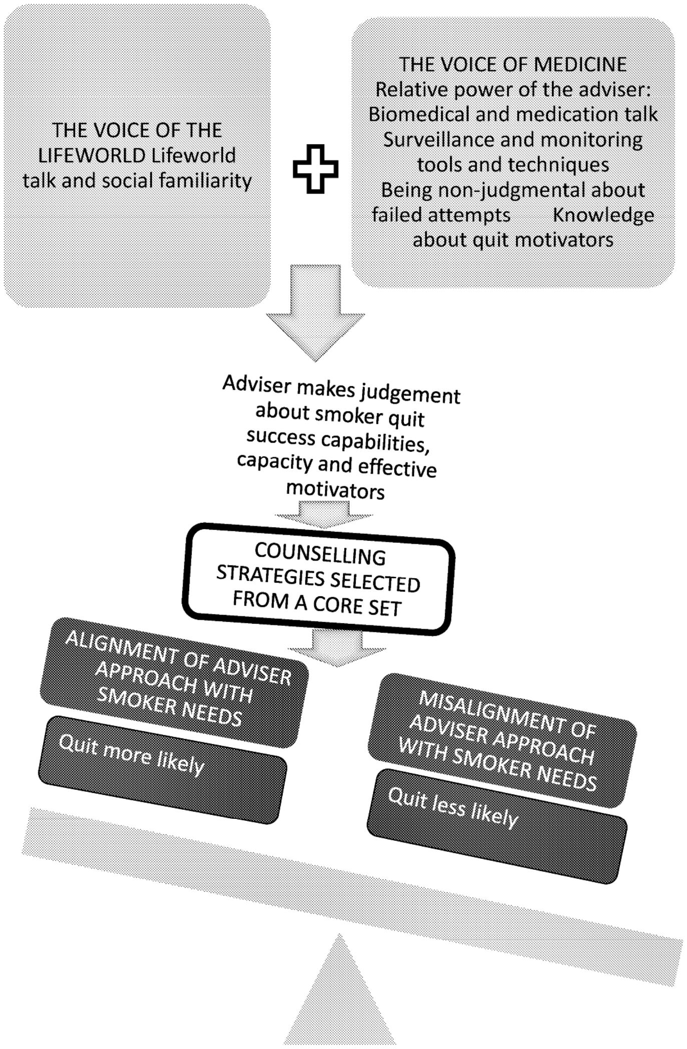

Figure 1: Tentative model of misalignment in the community pharmacy NHS Stop Smoking service consultation.

296x419mm (300 x 300 DPI)

BMJ Open

Determining counselling communication strategies associated with successful quits in the National Health Service community pharmacy Stop Smoking programme in East London: a focused ethnography using recorded consultations

Journal:	*BMJ Open*
Manuscript ID	bmjopen-2016-015664.R2
Article Type:	Research
Date Submitted by the Author:	02-Aug-2017
Complete List of Authors:	Rivas, Carol; University College London Social Science Research Unit; Queen Marys School of Medicine and Dentistry, Centre for Primary Care and Public Health<br>Sohanpal, Ratna; Queen Marys School of Medicine and Dentistry, Centre for Primary Care and Public Health<br>MacNeill, Virginia; University of Oxford<br>Steed, Liz; Queen Marys School of Medicine and Dentistry, Centre for Primary Care and Public Health<br>Edwards, Elizabeth; Queen Mary University of London, Centre for Primary Care and Public Health<br>Antao, Laurence; Queen Mary University of London<br>Griffiths, Chris; Queen Mary University of London<br>Eldridge, Sandra; Queen Mary University of London, Centre for Primary Care and Public Health<br>Taylor, Stephanie; Queen Mary University of London, Primary Care and Public Health<br>Walton, Robert; Queen Mary University of London
**Primary Subject Heading**:	Smoking and tobacco
Secondary Subject Heading:	Health services research, Public health, Qualitative research, Patient-centred medicine
Keywords:	Smoking cessation, Community Pharmacies, QUALITATIVE RESEARCH, Communication, Focused ethnography

SCHOLARONE™ Manuscripts

Determining counselling communication strategies associated with successful quits in the National Health Service community pharmacy Stop Smoking programme in East London: a focused ethnography using recorded consultations

Carol Rivas[12]

Ratna Sohanpal[2]

Virginia MacNeill[3]

Liz Steed[2]

Elizabeth Edwards[2]

Laurence Antao[2]

Chris Griffiths[2]

Sandra Eldridge[2]

Stephanie Taylor[2]

Robert Walton[2]

[1] SSRU, University College London, UK

[2]Centre for Primary Care and Public Health, Queen Mary University of London, London, UK

[3]University of Oxford, UK

Corresponding author:

Carol Rivas

Senior Researcher (Associate Professor)

SSRU

University College London

London, WC1H 0NR

Email: c.rivas@ucl.ac.uk

For peer review only

For peer review only - http://bmjopen.bmj.com/site/about/guidelines.xhtml

ABSTRACT

Objectives: To determine communication strategies associated with smoking cessation in the National Health Service community pharmacy Stop Smoking programme.

Setting: Eleven community pharmacies in three inner east London boroughs.

Participants: Nine Stop Smoking advisers and sixteen pairs of smokers who either quit or did not quit at four weeks, matched on gender, ethnicity, age and smoking intensity.

Method: 1-3 audio-recorded consultations between an adviser and each pair member over 5-6 weeks were analysed using a mixed method approach. First a content analysis was based on deductive coding drawn from a theme-oriented discourse analysis approach and the Roter Interaction Analysis System (RIAS). Core themes were identified through this quantification to explore in detail the qualitative differences and similarities between quitters and non-quitters.

Results: Quantitative analysis revealed advisers used a core set of counselling strategies that privileged the 'voice of medicine' and often omitted explicit motivational interviewing. Smokers tended to quit when these core strategies were augmented by: supportive talk; clear permission for smokers to seek *additional* support from the adviser between consultations; encouragement for smokers to use willpower. The thematic analysis highlighted the choices made by advisers as to which strategies to adopt and the impacts on smokers. The first theme 'Negotiating the smoker-adviser relationship' referred to adviser judgements about the likelihood the smoker would quit. The second theme 'Roles of the adviser and smoker in the quit attempt', focused on advisers' counselling strategies, while the third theme, 'Smoker and adviser misalignment on reasons for smoking, relapsing and quitting' concerned inconsistencies in the implementation of NCSCT training recommendations.

Discussion: Advisers in community pharmacies should use the advantages of their familiarity with smokers to ensure appropriate delivery of patient-centred counselling strategies, and reflect on the impact on their counselling of early judgments of smoker success.

Keywords: Smoking cessation, Community Pharmacies, Qualitative research, Focused ethnography, Communication

For peer review only - http://bmjopen.bmj.com/site/about/guidelines.xhtml

Strengths and limitations of this study

- To our knowledge this is the first study to consider verbal interactions in real world consultations between community pharmacy advisers and smokers in the NHS community pharmacy Stop Smoking programme
- 32 smokers from a larger sample were matched on demographic variables to provide 16 matched pairs of four-week quitters and non-quitters, enhancing confidence in findings.
- The findings may not be transferable to other pharmacies, particularly in less disadvantaged areas, or to over the counter community pharmacy interactions since the consultations took place in dedicated consultation rooms.
- Our study findings are limited to interactions during the consultation, as appropriate to the methods chosen, and we did not systematically explore previous quit attempts and their influence on results.

Introduction

UK community pharmacies have adopted public health and healthy living tasks that were previously the domain of the general practitioner.[1] These 'enhanced services' are embedded in UK health service policy and embraced by pharmaceutical professional bodies.[2-9] Community pharmacies have great potential to provide such health care, particularly for people from disadvantaged groups,[10] given their community setting, long opening hours, accessibility, familiarity and informality.[11-13] Pharmacy enhanced services therefore provide an opportunity to address health inequalities and may free general practice time for patients with more complex problems.[14]

The NHS Stop Smoking programme is one of the most frequently delivered enhanced community pharmacy services in England[15,16] and its effectiveness in 2015/16 measured by smoker self-reported 4-week quit rates was only slightly lower than for general practice (44% vs. 49%).[17] But neither general practice nor community pharmacies achieve the suggested optimal quit rate of 70%,[7] and there is considerable variation in rates within the same setting.[17] Moreover, the proportion of smokers enrolling on the Stop Smoking programme in community pharmacies rather than GP surgeries and other settings has remained stable since the programme began in 2006, falling between 18-21%, for example 20% in 2015/16.[7,17] This is despite government efforts to shift some of the GP workload to them.[7,15]

Enhanced services have necessitated a change in mind set for pharmacy staff with a move towards the person-centred care model currently advocated within health services.[17-26] Pharmacy staff tend to be inadequately trained in this model,[24] and may lack necessary consultation skills,[24,26] which may affect the growth of the Stop Smoking service in pharmacies. We therefore used a 'focused ethnography' approach[27,28] to explore counselling strategies used in consultations between pharmacy Stop Smoking advisers and smokers. Our primary aim was to use the findings to develop an intervention intended to improve both service uptake and quit rates in the community pharmacy Stop Smoking programme, as reported elsewhere.[29]

Methods

Focused ethnography

Using focused ethnography[27,28] we collected and analysed recordings of naturalistic (real world) consultations between pharmacy Stop Smoking advisers and smokers; recordings were made by the advisers themselves for the study. We considered verbal interactions that might affect smoker engagement, maintenance within the pharmacy programme and success in quitting.

For peer review only - http://bmjopen.bmj.com/site/about/guidelines.xhtml

In traditional ethnographic work, researchers immerse themselves in the social worlds of their participants to better understand the ways they interact with these worlds. They then generate textual or visual accounts of this for dissemination.[30] In focused ethnography, field visits are either much shorter, and selectively focussed, or replaced by audio- or video- or photo-recordings which may be undertaken with the researcher absent from the scene, as in our study. This approach is useful for answering a specific research question when traditional ethnography is impractical.[28]

Pharmacies and advisers

Consultations were recorded between November 2013 and May 2014 at community pharmacies contracted by Public Health England to provide the Stop Smoking programme in three inner East London boroughs (Newham, Tower Hamlets, the City and Hackney). These areas are economically disadvantaged compared with other areas of London and with England as a whole[31] and have relatively poor NHS Stop Smoking programme attendance.[13,15,16] We only included community pharmacy chains or independent single pharmacies, which are distinct from those in institutional settings such as NHS hospitals. Consultations had to take place in a private room. Only the adviser and smoker were present though sometimes the consultations were interrupted as they often took place in rooms that doubled as store rooms, which other staff needed to access

To select pharmacies, we used maximum variation sampling by borough, pharmacy size and length of time on the Stop Smoking programme. Within pharmacies, we invited any staff members certificated through the National Centre for Smoking Cessation and Training (NCSCT) programme, with a mixture of pharmacists and counter assistants therefore included in the study. We considered sampling for variation in years spent counselling for smoking cessation and in certification level in the NCSCT but there was too much missing data for this to be useful. In any case our focus was on the interaction between smoker and adviser per se. We visited pharmacies on spec or by appointment, explained the study, and if relevant took consent, and trained them in research methods and governance, so that they could take smoker service user consent and record, securely store and transfer their own consultations to the research team. This meant the natural rhythm of the service was not disrupted. Ethical approval for the study was obtained from the NRES Committee South Central, Berkshire B (reference number 13/SC/0189).

Smokers

All adult smokers recruited onto the NHS Stop Smoking programme during the course of the study were eligible and invited to join the study by the pharmacy adviser. Smokers were designated as having quit if they self-reported a smoke-free status at 4 weeks after their set quit date and provided an adviser with an expired carbon monoxide (CO) reading of less than 7 parts per million using a CO monitor. Smokers lost to follow up at four weeks were considered still smoking following the Russell criteria.[32]

Data collection

Community pharmacy NHS Stop Smoking programme consultations and prescriptions for medication may continue for up to 12 weeks with formal consultations weekly or two-weekly and informal drop-ins between these times as needed.[7,33] The core period for the programme ends at five to six weeks which tends to equate with the fourth week after the quit date, usually set at the first consultation.[7,33]

In line with our focused ethnography approach Stop Smoking advisers consented eligible smokers and selectively audiorecorded:

For peer review only - http://bmjopen.bmj.com/site/about/guidelines.xhtml

1. their first consultation, which tends to set the parameters for the remaining sessions, includes the smoker setting a proximal quit date, and is usually up to three times as long as subsequent sessions (averaging 15 minutes vs 5 minutes);[34]
2. the consultation at 2 weeks post proposed quit date where the adviser explores and attempts to solve any issues in the quit attempt;
3. the consultation four weeks post proposed quit date at which the smoker's quit status is formally designated (as the 4-week quit status) for Public Health England audit statistics and pharmacy remuneration[35] (see Sohanpal et al [2016][36] for a discussion of remuneration in relation to our larger study).

We call these weeks 1, 2 and 4 for simplicity. Recordings were anonymised and transcribed as they were received. Pseudo-anonymised metadata (such as demographic data and smoking intensity for the smokers, and training and demographic data for the advisers) were entered with anonymised recordings and transcripts onto a secure clinical trials database.

Matched pairs

We refer to quitters and non-quitters throughout this paper but in consultations 1 and 2 this is a retrospective designation for eventual quitters and non-quitters. We matched 16 pairs of 4-week quitters and non-quitters (i.e. 32 smokers) on gender, ethnicity, age (according to UK Office for National Statistics [ONS] age bands), and smoking intensity (fewer than 10 cigarettes a day, 10-20 a day, more than 20 a day). We had initially aimed for 20 matched pairs but obtained saturation at this lower number and so stopped data collection at this point.

Consultations analysis

We used a mixed methods analysis approach, in which themes were identified deductively and inductively and then quantified (Figure 1). First we developed deductive themes from a list of the key linguistic devices used in theme-oriented discourse analysis,[37] which considers how language use constructs professional practice, and the structuring of the medical consultation in the Roter Interaction Analysis System (RIAS),[38] which is a widely used method for coding and quantitatively analysing the conversational strategies used structurally in medical consultations. Our approach thus has its origins in psycholinguistics and its purpose is to look at what talk itself is doing as constitutive of social action, rather than as representational of inner psychological states, behaviours, beliefs or attitudes.[37]

The core team then applied the deductive themes to the consultation transcripts and also looked for emergent (inductive) themes[39,40]. immersing themselves in the data, reading and re-reading each consultation transcript and meeting to discuss themes. CR led development of themes from the data. The team constantly compared deductive and emerging inductive themes with each other and also compared data within themes to ensure each emergent theme was discrete from all other themes and representative of the relevant data.[39,40] CR operationalised the themes, for a second researcher (RS) to code 20% of the data independently. Differences were reconciled by discussion, until a Cohen's kappa statistic of 76% was obtained.

Once themes were determined, a quantitative summary of these was developed. First, for each theme we quantified the number of smokers whose consultations included the theme (A of Table 1). This suggested what strategies were typically used in the counselling sessions (thus a core set of strategies). We also calculated the average frequency of use of a theme within consultations in which it was mentioned (D of Table 1) by dividing the total number of occurrences of a theme across all consultations (column C of Table 1) by the number of consultations in which it occurred (column B of Table 1). We only counted one occurrence in each 'turn' the smoker or adviser took to talk

For peer review only - http://bmjopen.bmj.com/site/about/guidelines.xhtml

(where a conversation is made up of lots of turns at talking). This gave us a measure of the relative importance of each theme; a theme with an average frequency of 1 may be considered less important overall than one with an average frequency of 4, that is, it may be used only in very specific circumstance or idiosyncratically. To obtain the frequency measure we divided by the number of consultations with a mention of the theme rather than all consultations because we wanted a measure that indicated repetitions. We could have provided an alternative measure of the relative bias towards a theme within consultations by dividing the number of mentions of all themes in each consultation by the number of mentions of each specific theme. However this measure would be sensitive to the absence of other themes because they were not relevant (for example because the three weeks we recorded had different foci) or because they had been covered outside of the recording. For similar reasons we did not quantify the number of words used for each theme.

With all quantitative measures (as with the qualitative analysis) we looked for similarities and noted any differences between eventual quitters and non-quitters. We used the quantitative measures to give us a broad picture of the consultations and themes so that we could determine which to explore in more detail qualitatively. We focussed in on themes that were associated with quit success or lack of success, and that were of high or moderate frequency of use across consultations or that appeared to be core to the sessions. We also considered strategies that were rarely used but that might be important for service improvement. This led to a list of key themes, which we report on in more detail in the next section. Given the small numbers involved, tests of significance are not reported here.

Findings

Participants

Thirty-nine percent (11/28) of recruited advisers provided 158 audiorecordings from 53 smokers; two further pharmacies dropped out due to lack of time. Considering only the 16 matched pairs, these were seen by 9 of the 11 advisers, with a median of three smokers each (range 1-7). Table 2 compares baseline characteristics of the matched pairs to the total sample . Whilst women were under-represented in the matched pairs, other characteristics related to cessation outcome were broadly similar. This includes medication use; though we did not monitor for this systematically, the transcripts indicate that similar numbers of quitters and non-quitters were on vareniciline, for example. Identification (ID) numbers in the tables are also used to attribute extracts from the data in the narrative text. Most advisers in the total group were male (89%) and from pharmacies in City and Hackney (89%). Sixty-seven percent of advisers were Indian, and 11% were from other black and minority ethnic (BME) groups, with missing ethnicity data for one. Importantly, findings were similar whether the adviser was a pharmacist or trained counter assistant. With one exception, there was no indication of a lesser authority in counter assistants since they, like the pharmacists, had received special training in smoking cessation counselling. The exception had only recently been trained and was being shadowed by the pharmacist.. The small number of advisers in the study precluded analysis of the effect of other variables on the consultation. In any case our analysis was focussed on what talk itself does, and was not intended to explore these effects.

Themes

Thematic analysis reached saturation, and revealed three overarching themes in the data,

- Negotiating the smoker-adviser relationship
- The roles of the adviser and the smoker in the quit attempt
- Smoker and adviser misalignment on reasons for smoking, relapsing and quitting

For peer review only - http://bmjopen.bmj.com/site/about/guidelines.xhtml

These were made up primarily of the subthemes developed deductively from psycholinguistics as described in the methods. Table 2 provides a quantitative overview of the themes, and selected subthemes based primarily on the RIAS and theme-oriented discourse analysis, which we focus on in this paper, and compares quitters and non-quitters. We follow this with a narrative description of themes; extracts are identified by smoker (P), adviser (A) and consultation week (W) while the quantifiers most, common or many, occasionally or some, and a few or rarely, refer to a theme occurrence of 75%+, 50-74%, 25-49% and less than 25%. The first consultation should consider smoker tobacco use and motivation to quit (some advisers used a questionnaire type script) and subsequent ones build on this. Here we focus on what the talk does within any adviser-smoker interaction and the association of interaction strategies per se with quitting or not, hence aggregating the data across consultations, whilst mindful of which consultations they represent. We consider the data by consultation sequence in further analyses to be reported elsewhere, which support the findings of this paper.

From the quantitative data we determined that the core set of strategies used by advisers (occurring in 50% or more of smokers) were: praise; biomedical talk and advice; mention of the side effects of medication; collaborative talk; the use of props and strategies to cope with the urge to smoke; and the use of monitoring and surveillance talk. Significantly, explicit use of motivational talk is not included in this core set.[41] Further strategies were added to the core set in eventual quitters: talk on the importance of support; open door talk; the need for willpower. Managing expectations formed part of the core set for non-quitters only. These themes are discussed in more detail below.

For peer review only

For peer review only - http://bmjopen.bmj.com/site/about/guidelines.xhtml

THEME#	A: NUMBER OF SMOKERS WITH THEME		B: NUMBER OF CONSULTATIONS WITH THEME (%)		C: TOTAL NUMBER OF OCCURRENCES OVERALL	D: MEAN FREQUENCY OF MENTIONS IN CONSULTATIONS IN WHICH THEME APPEARS (i.e columns C/B)
	NUMBER OF QUITTERS WITH THEME (% OUT OF n=16)	NUMBER OF NON-QUITTERS WITH THEME (% OUT OF n=16)	QUITTERS OUT OF 30 CONSULTATIONS, n (%)	NON-QUITTERS OUT OF 29 CONSULTATIONS n (%)	QUITTERS, NON-QUITTERS	QUITTERS, NON-QUITTERS
**Negotiating the smoker-adviser relationship**						
Lifeworld talk	10 (63%)	9 (56%)	19 (63%)	18 (62%)	75,45	3.95, 2.5
Adviser being non-judgmental	4 (25%)	3 (19%)	6 (20%)	5 (17%)	9, 10	1.50, 2.00
Adviser praise	10 (63%)	9 (56%)	22 (73%)	20 (69%)	64, 53	2.91, 2.65
**The roles of the adviser and the smoker in the quit attempt**						
Receiving biomedical information and advice	14 (88%)	11 (69%)	25 (83%)	16 (55%)	106, 48	4.24, 3.00
Importance of support	8 (50%)	5 (31%)	14 (47%)	6 (21%)	31, 13	2.21, 2.17
Mentioning side effects of medication	9 (56%)	8 (50%)	12 (40%)	11 (38%)	34, 26	2.83, 2.36
Adviser mentioning relapse	4 (25%)	3 (19%)	9 (30%)	6 (21%)	12, 6	1.33, 1.00
Motivational talk	5 (31%)	5 (31%)	15 (50%)	12 (41%)	37, 34	2.47, 2.83
Confidence in being able to quit	3 (19%)	1 (6%)	8 (27%)	2 (7%)	8, 3	1.00, 1.50
Need for willpower	8 (50%)	7 (44%)	9 (30%)	13 (45%)	17, 18	1.89, 1.38
Collaborative talk (e.g. ‘we’)	10 (63%)	8 (50%)	20 (67%)	15 (52%)	67, 51	3.35, 3.40

For peer review only - http://bmjopen.bmj.com/site/about/guidelines.xhtml

Ownership of the quit	5 (31%)	4 (24%)	8 (27%)	5 (17%)	16, 6	2.00, 1.20
Managing smoker expectations of the programme	5 (31%)	9 (56%)	9 (30%)	11 (38%)	19, 30	2.11, 2.73
The use of quit props and strategies	11 (69%)	10 (63%)	22 (73%)	22 (76%)	73, 39	3.32, 1.77
'Open door' talk (invitations to informal consultations between the formal ones)	9 (56%)	7 (44%)	10 (33%)	8 (28%)	15, 12	1.50, 1.50
Monitoring and surveillance	14 (88%)	15 (94%)	21 (70%)	17 (59%)	38, 38	1.81, 2.24
**Smoker and adviser misalignment on reasons for smoking, relapsing and quitting**						
Smoker explanations for smoking						
Stress	6 (38%)	9 (56%)	8 (27%)	13 (45%)	19, 17	2.38, 1.31
Social factors	4 (25%)	3 (19%)	6 (20%)	4 (14%)	8, 6	1.33, 1.50
Financial reasons	5 (31%)	5 (31%)	5 (17%)	6 (21%)	6, 8	1.20, 1.33
Health problem	8 (50%)	11 (69%)	8 (27%)	13 (45%)	19, 32	2.38, 2.46
Smoker explanations for wanting to quit						
Quitting for families (including children)	5 (31%)	3 (19%)	6 (20%)	3 (10%)	15, 3	2.50, 1.00
Obligations to self and others	2 (13%)	1 (6%)	4 (13%)	1 (3%)	6, 1	1.50, 1.00
Negative feelings towards smoking	1 (6%)	3 (19%)	1 (3%)	5 (17%)	1, 5	1.00, 1.00
Negative impact of smoking on appearance (e.g. Ageing effects) or identity (e.g. Smelly)	1 (6%)	3 (19%)	2 (7%)	5 (17%)	2, 8	1.00, 1.60
Adviser-suggested motivator for smoker to quit						
Financial	4 (25%)	6 (38%)	10 (33%)	8 (28$)	14, 9	1.40, 1.13

For peer review only - http://bmjopen.bmj.com/site/about/guidelines.xhtml

Health scare tactics	4 (25%)	3 (19%)	5 (17%)	3 (10%)	8, 5	1.60, 1.67

Themes shown here have been selected from a larger pool for this paper.

For peer review only

For peer review only - http://bmjopen.bmj.com/site/about/guidelines.xhtml

Table 1: Quantitative comparisons between quitters and non-quitters in: A) number of quitters and non-quitters for which each theme was identified (second and third columns); B) number of consultations overall in which each theme was identified (middle columns); C) total number of occurrences in all consultations (penultimate column); D) relative frequency with which themes were used in individual consultations (final column). In the narrative text we only consider values for A and D.

VARIABLE	QUITTERS (16)	NON-QUITTERS (16; 6 LTF)	ALL (53; 9 LTF)
FEMALE	3 (19%)	3 (19%)	18 (34%)
AGE (MEDIAN, RANGE)	47 (26-58)	41 (28-59)	41.13 (18-67)
OCCUPATION			
MANAGERIAL AND PROFESSIONAL	2 (13%)	0	5 (9%)
UNEMPLOYED	10 (63%)	10 (63%)	25 (47%)
ROUTINE MANUAL	3 (19%)	3 (19%)	8 (15%)
STUDENT	0	0	1 (2%)
INTERMEDIATE	0	2 (13%)	7 (13%)
RETIRED	0	0	1 (2%)
?NO ANSWER	1 (6%)	4 (25%)	6 (11%)
ETHNICITY			
WHITE BRITISH	11 (69%)	9 (56%)	30 (57%)
SOUTH ASIAN	3 (19%)	3 (19%)	8 (15%)
OTHER WHITE	2 (13%)	3 (19%)	10 (19%)
BLACK AFRICAN	0	0	1 (2%)
BLACK CARIBBEAN	0	0	1 (2%)
MISSING DATA	0	1 (6%)	2 (4%)
CIGARETTES SMOKED			
40	1 (6%)	0	1 (2%)
30	2 (13%)	2 (13%)	5 (9%)
25	3 (19%)	3 (19%)	8 (15%)
20	7 (44%)	9 (56%)	25 (47%)
15	1 (6%)	2 (13%)	5 (9%)
<15	2 (13%)	1 (6%)	9 (17%)
PCT			
TOWER HAMLETS	2 (13%)	5	12 (23%)
CITY AND HACKNEY	14 (88%)	10	38 (72%)
NEWHAM	0	0	3 (6%)

Table 2: Smoker demographic and smoking data summaries for total number sampled and for the matched pair groups (LTF = lost to follow-up).

For peer review only - http://bmjopen.bmj.com/site/about/guidelines.xhtml

1. Negotiating the smoker-adviser relationship

Lifeworld talk was common across the groups, though occurring in slightly more eventual quitters and with more occurrences within their consultations. This is talk about a person's experience of life with all its contextual and situational nuances.[42-45] Through lifeworld talk, the advisers gained a shared understanding with the smoker of the context of the smoker's quit attempt and the individual difficulties they faced. The adviser could then discuss approaches to quitting that took into account such things as the stress that the smoker in the following extract describes.

.

> A: So whilst you're on Champix and you left work and you're a bit stressed did you say?
> S: Yeah, because there's many changes in the work you remember when you told me in the beginning, like try to, don't change the work and the things. But they change our store manager, my manager is leaving. The work we used to do, six persons now going to be in two persons, oh my god. It's just,
> A: So you're a bit more stressed at work.
> S: Terrible. It's terrible.
> A: So you came home and you're a bit stressed still, so you felt like you wanted a cigarette? But you didn't have a cigarette? (P807-A81-W4, quit)

The advisers made it clear to around one quarter of smokers that they were not going to judge them for any lapses and failures, as a way to motivate them to continue the programme, illustrated in the following extract. This is in line with good practice.

> A: Fantastic. So again, my idea is to mentor you and support you and that's what it's about, yeah? I'm not here to tell you off. It's about where you're finding it hard and what we need to work together to find a solution for you. (P107-A1-W1, quit)

Praise was common across groups, but with marked qualitative differences. Quitters received limited and brief praise. By contrast, eventual non-quitters were given extensive (extreme formulation[46,47]) praise even when they had not achieved the NCSCT-recommended 'not a puff', as below.

> S: Well, it's actually helped a lot, so I'm only smoking around ten.
> A: Ten plus? Ten to fifteen?
> S: Ten plus.
> A: That's fantastic. In the space of six, seven days you've cut down by over half. (P4-A1-W2, not quit)

2. The roles of the adviser and the smoker in the quit attempt

Biomedical information and advice was imparted across the groups but quitter consultations were most heavy with it. This may mean they were more open to biomedical talk or that the adviser got an impression that they were, or it may be an incidental finding. Side effects of the medication and withdrawal symptoms from stopping smoking, including weight gain, were discussed with many smokers. The possibility of relapse was discussed with only 25% of quitters and 19% of non-quitters. There was some compensation in the degree to which the use of strategies to deal with cravings (hence *risk* of relapse) was commonly encouraged (see extract later in this section).

Advisers made attempts to manage many of the eventual non-quitters' expectations of the programme as well as emphasising their required commitment. Both strategies were undertaken only occasionally with quitters. Many quitters but only some non-quitters were told of the

For peer review only - http://bmjopen.bmj.com/site/about/guidelines.xhtml

importance of advisers' counselling support. Advisers' motivational talk was occasional in both groups, and did not form part of the core set of strategies and advisers only rarely asked for (and therefore got) expressions of confidence in being able to quit (3 quitters over 8 consultations vs 1 non-quitter over 2 consultations), a specific element of motivational interviewing. The need for the smoker to draw on their own willpower to quit was considered in most quitters but only some non-quitters, and the necessary balance between adviser support and more personal willpower or motivation was only discussed with one non-quitter (in the following extract) and one quitter.

> S: I've got a photograph of me taken when I was in intensive care in hospital, I should take a look at that every time I want a cigarette.
> A: Perfect! You need some motivation.
> S: I don't want to end up like that again.
> A: Exactly, you need that motivation. And if there's anyone else that can help you also…
> S: My mum, yeah, she's very supportive.
> A: Sure. You need that support as well. But you've got us as well because you'll be coming here every week. (P806-A81-W1, not quit)

Collaborative talk (such as the use of the phrase "we will succeed"), a supportive stratagem, was common in both groups. The related topic 'smoker's ownership of the quit' was rarely mentioned in non-quitter consultations and only slightly more so in quitters'. Some messages were implied rather than explicit. The use of props and strategies to deal with cravings requires some ownership and self-management of the quit and was recommended at least once in a majority of smokers and consultations, though more frequently discussed *within* quitter consultations. Many different props and strategies were suggested, with the following only one example.

> A: I want you to make sure you start off in the morning; you've got your bottle of 1.5 litres to 2 litres. Your aim, you start in the morning, you finish at the end of the day. Every time you want a cigarette, you're going to drink the water. You're going to complete it. (P101-A11-W1, not quit).

The advisers invited many eventual non-quitters to drop into the pharmacy for support and advice any time the shop was open, a particular advantage that pharmacies have over other healthcare settings. Such 'open door' talk, offering a more informal variant of adviser support, was said to fewer eventual non-quitters and was less successful for these.

In most smokers, the CO monitor was depicted by the adviser as a monitoring or surveillance tool that was used to check the smoker's progress and even catch the smoker out:

> A: Well, it's good that you're here, we will help you in any way we can. What I ask you every week is if I can take a reading from you. This is called a CO monitor and this machine measures the amount of carbon monoxide inside of your system. And I call this my lie detector. I'm not going to say that you're going to lie to me, but if you do come in and I say have you had any cigarettes, and you say no, I haven't had any, this will tell me the truth. (P7-A1-W1, quit)

3. *Smoker and adviser misalignment on reasons for smoking, relapsing and quitting*

An actual health problem or worry about getting one was cited as reason to quit by more than half the smokers and was invoked slightly more commonly by non-quitters. There was an inverse relationship between this and adviser use of health scare tactics to motivate the smoker, though these tactics were relatively uncommon. Financial reasons for wanting to quit were cited by just

For peer review only - http://bmjopen.bmj.com/site/about/guidelines.xhtml

under one third of smokers. However the advisers tried to motivate the smokers with talk of financial savings on 23 separate occasions. Initial misalignment is demonstrated in the following extract for example; though there is evidence of subsequent 'repair' of this at the end of the extract our focus in the current analysis was on whether misalignment arose at all.

> S: I mean I don't want to die. I don't want to have a stroke.
> A: So that's your main impulse now and more likely you'll succeed because of that.
> S: I don't want to end up like that…
> A: The other reasons why maybe you feel I'll give up now, maybe save a bit of money or whatever before…
> S: That's the backburner, that's nothing to do…
> A: Exactly. Was that the reasons before?
> S: No.
> A: What were the reasons before that you wanted to stop smoking?
> S: Smell; I don't like the smell. I've got this thing, when I eat, people smoke around me, I don't like it, I don't like the smell of it when I'm eating. People light one up when you're having something to eat and oh, it's disgusting, I don't like the smell. (P806-A81-W1, not quit)

Quitters were twice as likely to mention their families (including children) as motivation though under a third did so. Other reasons for smoking were cited relatively infrequently. Notably the misalignment or mismatch between smoker declaration of health as motivation to quit and adviser comments on health problems caused by smoking was more likely in non-quitters, and advisers were also less likely to mention motivational factors at all to eventual non-quitters. In quitters' consultations, advisers were likely to mention motivators, especially financial, even when the smoker did not.

Discussion

We applied rigorous and systematic analytical methods to a sample of audiorecordings of real world community pharmacy adviser-smoker NHS Stop Smoking consultations with the premise that the community pharmacy Stop Smoking programme should be person-centred. Specifically, we explored associations of thematic patterns in the verbal interactions with 4-week quit success. Although there are a number of ethnographic studies of client-pharmacy staff communication processes or interactions[24,38,48-71] there is none on stopping smoking in the community. Our overall findings accord with those of Pilnick's study[49-51] in a UK hospital paediatric outpatient clinic pharmacy, the most comparable study to ours, which focussed on advice-giving in the counselling role, but we provide rich new data.

There were some clinically potentially significant omissions across the data. For example talk about relapse was rare, though there was some compensation in the degree to which the use of strategies to deal with cravings was frequently encouraged and the biomedical problems of quitting often explored. We found many examples of good practice, including significant evidence of lifeworld talk,[42,43] which was common across the two groups. Mishler,[43] applying the Habermas theory of Communicative Action[72] to medical encounters, itself derived from Husserl's phenomenological concept of consciousness and subjectivity,[44] showed how this enables person-centred support. It provides the pharmacy advisers with the smokers' own nuanced and contextualised[73-81] accounts of their personal daily lives, in which they manage their quit attempts. When the adviser uses these to shape their recommendations, advice and medical support, adviser consultations are more likely to be effective.[44,82] The occurrence of lifeworld talk in our data suggests that the community setting and the advisers' social familiarity[81] with the smokers as customers enables such smoker-centred

For peer review only - http://bmjopen.bmj.com/site/about/guidelines.xhtml

care, in a way that is potentially greater than is possible in GP consultations. This supports other research suggesting that community pharmacies are ideally placed for smoking cessation and other health behaviour change tasks.[10-13]

Nonetheless, the core set of strategies common across both quitter and non-quitter groups focussed on the practical and the medical, biomedical talk and advice (the 'voice of medicine'[42,43]). This was certainly an important part of consultations, and appropriate given the pharmacy staff member's role as *adviser* and the need for such talk in the first consultation in particular. But unlike biomedical talk, lifeworld talk was not included in this core set, in other words it was less consistently used than. We found, from more detailed analysis, that differences between smokers in the way lifeworld talk was used could be related to quit success or failure.

It appeared that advisers used lifeworld talk to make judgments about likely smoker quit success. This then compromised its effective use. We found this affected non-quitters disproportionately, and this seemed due to the adviser focussing on possible problems in those they judged as less likely to quit, and making assumptions about reasons the smoker found it hard to quit. This counselling approach was not used in those who went on to quit. Our data show that in this way, pre-judgements led to three sources of misalignment with, or confusion in, the smokers who did not quit. First, advisers tended to offer casual 'open door' drop-in support between consultations more often to eventual non-quitters, perhaps because they perceived they needed more support. Bu these offers were not clear or explicit in their purpose and appear to have simply confused smokers as to when they were meant to attend. Second, praise and willpower talk were common in both quitters and non-quitters but the way they were communicated was often poorly matched to smoker effort. Extreme statements made in praise of non-quitters when they were still smoking created cognitive dissonance. Third, the advisers drew on financial and health benefits as motivators for quitting in those who then failed to quit and had cited other reasons for wanting to quit. The misalignment between the smoker's reasons for smoking or wanting to quit, and the adviser's perceptions of what these were, would result in the adviser setting inappropriate goals for the smoker.[72-82]

These misalignments may therefore explain why a smoker's sharing of their lifeworld was not always associated with quit success. This interpretation of the data is supported by similar findings in our separate analysis of adviser interviews, with some advisers explicitly stating that they emphasised particular strategies in those smokers they considered would find it harder to quit, to try to help them.[36] Misalignments push interactants 'out of sync' with each other, making it hard for them to achieve a common goal. Nyugen considers how misalignments in pharmacy counselling need to be delicately managed in order not to affect the tenor of the rest of the conversation.[62] Misalignments were noted by Pilnick[49-51] as problematizing the adviser's advice giving but we have shown how they develop to create issues in the adviser's cognitive development of what that advice might be as well as how their counselling support should be configured.

Our data also show that the advisers drew on sometimes apparently contradictory strategies to induce a quit. For example, they often combined reassurance of a non-judgmental approach with the use of the CO monitor as a surveillance tool. While this seems to have been successful, biomedical talk and advice, talk of being non-judgmental, monitoring and surveillance, and the advisers' use of authority and expertise to prejudge likely smoker success, are all premised on a power differential between adviser and smoker and may therefore appear to sit in dialectical tension with the development of shared understandings and social familiarity from the community setting. Social familiarity and power differentials are not mutually exclusive but they are less likely to co-exist, the greater the difference in social status between two interactants.[72,78,81,83,84] Thus the

For peer review only - http://bmjopen.bmj.com/site/about/guidelines.xhtml

pharmacy community setting provides particular communication issues that are less likely to occur in encounters with doctors and medical specialists. Social familiarity is shown in our data to have the potential to enhance quit success and power differentials to compromise this, and the best outcomes might be achieved when advisers strike a balance between the two, optimising the benefits of the community pharmacy setting. Figure 2 demonstrates this. Considering recommendations for pharmacy adviser smoking cessation care, we found successful quits to be more likely when there was more smoker-centred care with counselling plans that were based on the smokers' own lifeworlds, and that matched strategies such as praise to the smokers' actual rather than predicted behaviours.

Strengths and limitations

Our study contributes to current knowledge and practice with novel data from a pharmacy service that has not previously been subject to ethnographic study. As this was a qualitative study we considered data from a small and varied sample of participating staff members, smokers and community pharmacies, adding to richness of the findings. Since we were comparing two groups, we matched them on relevant variables to increase the dependability of our analysis. We achieved theme saturation within this matched pair design, with adequate agreement between coders, group data sessions and consideration of negative cases in the data adding to credibility and dependability of findings. Themes reported in this analysis correspond with some of the themes developed separately from semi-structured qualitative interviews with advisers and smokers from the same study.[36] Our study has enabled us to identify key elements of talk and can be used to strengthen consultations and make them more effective.

Our study also has limitations. We were not successful in recruiting pharmacy chains and make no claims to transferability of the findings to other pharmacies, particularly those in more affluent areas. Further, it may not be possible to extrapolate findings from these consultations, which were undertaken in a private room, to over the counter community pharmacy interactions. In collecting naturalistic qualitative data, our aim was rather to provide in depth exploration of communication practices. This was usefully achieved. A number of contextual and demographic variables are known to affect pharmacist-smoker communication and quit successes.[85] We were unable to explore these in our small sample. We did not case match by adviser ethnicity or other adviser variables as this would have required oversampling for our specific research question, which was not intended to explore this, but this might be a topic for different research. However we systematically looked for adviser-specific patterns and found that our analysis held irrespective of adviser. Participants were not blinded to the nature of the research and had a potential vested interest in successful quits, creating a possible performance effect. However the unobtrusive data collection method will have reduced this risk with smokers and the effect would have been the same for quitters and non-quitters. There may have been an intrinsic difference between the quitters and non-quitters before they even entered the consultation room and our study was not designed to explore this. For example, although baseline characteristics known to be related to quitting, such as gender and age were taken into account we did not systematically collect data on other potentially significant variables such as number of previous quit attempts or medication use. Our thematic analysis suggests this would have been useful. The quitters may have been successful irrespective of consultation features; the availability of support itself may be sufficient.[41,85]

Clinical implications

Advisers had all received National Centre for Smoking Cessation Training (NCSCT), which focuses on counselling skills but does not consider implementation in practice. Nor does it cover initial smoker engagement and retention in the service. Moreover, whilst NCSCT training has been shown to be

For peer review only - http://bmjopen.bmj.com/site/about/guidelines.xhtml

effective,[86] we previously identified through semi-structured interviews that advisers lacked confidence when implementing the training in practice.[36] They wished for more support that built upon and reinforced many of the behaviour change techniques learnt through the NCSCT scheme, and that also addressed client-centred consultation skills.[36] These issues may part explain the advisers' inconsistencies in the delivery of and the content of the consultations. To improve their practice, advisers might reflect on their use of the considerable lifeworld talk generated by smokers through their social familiarity with the pharmacy advisers. Lifeworld talk should be used to modify adviser counselling to suit key external psychosocial factors, namely the *context* within which the quit attempt is made and the related *motivators* to quitting. Our data suggest that currently advisers may instead modify the consultation to suit their assumptions about internal psychological factors such as the *smoker's capacity to quit or motivation for quitting*.[41] In this way advisers sometimes develop the consultation in ways that, while well-meaningly intended, run the risk of being counter-productive. For smokers judged by them as less likely to quit this may include inappropriately overemphasising some support strategies (such as praise, managing expectations) and underplaying others (such as the need for willpower and the importance of adviser support). Our data illustrate the potential for misalignment that this creates. Moreover, the exaggerated praise we saw in non-quitter reductions in smoking conflicts with the 'not a puff' rule recommended by the NCSCT. Advisers should use findings from our study to clarify where they might usefully capitalise on the social closeness they seem to share with the smokers they see, with more appropriate use of lifeworld talk and more use of smoker-declared motivators during motivational interviewing.

Recommendations for research

Our study only considers short-term outcomes, something we are addressing with further research. The analyses have been used to inform the development and content of our training intervention for pharmacy Stop Smoking advisers, evaluated in a cluster randomised trial (grant number RP-PG-0609-10181). This intervention complements NCTSC training and is not intended to replace it. We are currently exploring the micro-detail of the talk in the consultations using conversation analysis with a view to further dissemination and use. Our study could be replicated in less disadvantaged areas or in specific minority groups.

4487 words

Acknowledgments The authors thank participants and their community pharmacies. This paper presents independent research funded by the National Institute for Health Research (NIHR) under its Programme Grants for Applied Research Programme. The views expressed are those of the authors and not necessarily those of the NHS, the NIHR or the Department of Health.

Contributors RW conceived the study. CR undertook the analysis and wrote the manuscript. RS, LA and VM were involved in data collection. RS, LS and RW contributed to the data analysis and interpretation. VM, LS, LA, EE, CG, ST, SE and RW were involved in revising the article critically and contributing important intellectual content. RW is the guarantor.

Competing interests None.

Funding: NIHR Programme grant RP-PG-0609-10181

References

For peer review only - http://bmjopen.bmj.com/site/about/guidelines.xhtml

References

1. Gidman W, Ward P, McGregor L. Understanding public trust in services provided by community pharmacists relative to those provided by general practitioners: a qualitative study. *BMJ Open* 2012;2:3 e000939 doi:10.1136/bmjopen-2012-000939
2. The Royal Pharmaceutical Society of Great Britain. *Pharmacy in a New Age: The New Horizon.* London: The Royal Pharmaceutical Society of Great Britain, 2006.
3. Department of Health. *Pharmacy in England: Building on strengths – delivering the future.* London: The Stationery Office, 2008.
4. Department of Health. *Speech by the Rt Hon Alan Johnson MP, Secretary of State for Health NHS Confederation Primary Care Network,* 2008 webarchive.nationalarchives.gov.uk/+/www.dh.gov.uk/en/MediaCentre/Speeches/Speecheslist/DH_083369 (accessed 21 December 2015)
5. Royal Pharmaceutical Society. *The Changing Face of Pharmacy,* https://www.rpharms.com/public-affairs-pdfs/rps-changing-face-of-pharmacy-booklet.pdf (accessed 9 January 2016)
6. NHS England. *Improving health and patient care through community pharmacy - a call to action,* 2013. http://www.england.nhs.uk/wp-content/uploads/2013/12/community-pharmacycta.pdf (accessed 24 Feb 2015)
7. NHS Employers. *Pharmacy-based stop smoking services: optimising commissioning*. London: NHS Employers, 2009.
8. Pharmaceutical Services Negotiating Committee, *The community pharmacy: A guide for general practitioners and practice staff.* July 2013.
9. NHS Confederation. *Health on the high street. Rethinking the role of community pharmacy,* London, 2013. www.nhsconfed.org/Publications/reports/Pages/Health-on-high-street-rethinking-community-pharmacy.aspx (accessed 24 Feb 2015)
10. National Pharmacy Association. *Pharmacy Health Trainers Resource*, 2009.
11. Fajemisin F. *Community Pharmacy and Public Health. Solutions for Public Health*, 2013. URL: http://www.sph.nhs.uk/sph-documents/community-pharmacy-and-public-health-finalreport/?searchterm=community pharmacy (accessed 21 Dec 2015)
12. Hametz I, Hodgkiss F, Sheehy C. *Review of the community pharmacy public health service for smoking cessation and emergency hormonal contraception*. Scottish Government, Edinburgh; 2011
13. Todd A, Copeland A, Husband A, Kasim A, Barbra C. The positive pharmacy care law: an area-level analysis of the relationship between community pharmacy distribution, urbanity and social deprivation in England, *BMJ Open* 2014;4:8 e005764 doi:10.1136/bmjopen-2014-005764
14. Ali A, Wright N, Rae M. *Addressing Health Inequalities – A guide for general practitioners*. Royal College of General Practitioners Health Inequalities Standing Group. London: RCGP, 2008
15. West R, May S, West M, Croghan E, McEwen A. Performance of English stop smoking services in first 10 years: analysis of service monitoring data. *BMJ* 2013;47:f4921.
16. Anon. Community pharmacy: At the heart of public health. Pharmaceutical Services Negotiating Committee; 2013
17. Lifestyle statistics, Health and Social Care Information Centre. *Statistics on NHS Stop Smoking Services: England, April 2015 – March 2016,* 2016
18. Bauld L, Hiscock R, Dobbie F, Aveyard P, Coleman T, Leonardi-Bee J, McEwen, A. English Stop-Smoking Services: One-Year Outcomes. *International Journal of Environmental Research and Public Health 2016;*13(12):1175. http://doi.org/10.3390/ijerph13121175

For peer review only - http://bmjopen.bmj.com/site/about/guidelines.xhtml

19. Bartlett EE, Grayson M, Barker R, Levine DM, Golden A, Libber S. The effects of physician communications skills on patient satisfaction; recall, and adherence. *J Chronic Dis.* 1984;37(9–10):755–764
20. Epstein RM, Street RL. The Values and Value of Patient-Centered Care. *Annals of Family Medicine* 2011;9(2):100-103. doi:10.1370/afm.1239
21. The Health Foundation. *Person-centred care made simple: What everyone should know about person-centred care*, The Health Foundation, London 2014.
22. Department of Health. Equity and Excellence: Liberating the NHS. Department of Health: London, July 2010
23. National Institute for Health and Care Excellence. *Falls in older people: assessing risk and prevention. NICE guidelines [CG161]*, June 2013
24. Royal Pharmaceutical Society Wales. *Your Care, Your Medicines: Pharmacy at the heart of patient-centred care*, 2015
25. Greenhill N, Anderson C, Avery A, Pilnick A. Analysis of pharmacist-patient communication using the Calgary-Cambridge guide. *Patient Education and Counseling* 2001; (83): 423-431
26. Salter C, Holland R, Harvey I, Henwood K. 'I haven't even phoned my doctor yet'. The advice giving role of the pharmacist during consultations for medication review with patients aged 80 or more: qualitative disclosure analysis. *BMJ* 2007; May 26;334(7603):1101
27. Stahlke Wall S. Focused Ethnography: A methodological adaptation for social research in emerging contexts. *Forum Qualitative Sozialforschung / Forum: Qualitative Social Research*, [S.l.], v. 16, n. 1, nov. 2014. ISSN 1438-5627. Available at: <http://www.qualitative-research.net/index.php/fqs/article/view/2182>. Date accessed: 14 may 2017.
28. Knoblauch H. Focused ethnography. *Forum Qualitative Sozialforschung / Forum: Qualitative Social Research, [S.l.]* 2005; 6(3), ISSN 1438-5627. Available at: <http://www.qualitative-research.net/index.php/fqs/article/view/20>. Date accessed: 29 apr. 2017.
29. Steed L, Sohanpal R, Yee W, Rivas C, Jumbe S, Chater A, Todd A, Edwards E, MacNeill V, Macfarlane F, Greenhalgh T, Griffiths C, Eldridge S, Taylor S, Walton R. Equipping community pharmacy workers as agents for health behaviour change: developing and testing a theory-based intervention. *BMJ Open* (accepted)
30. Wolf ZR Ethnography: the method. In Munhall PL (Ed) *Nursing Research – A Qualitative Perspective.* Fourth edition. 2005, Jones and Bartlett, Sudbury MA
31. Department for Communities and Local Government, *English indices of deprivation 2015*, Department for Communities and Local Government, London, 2015
32. West R, Hajek P, Stead L, Stapleton J. Outcome criteria in smoking cessation trials: proposal for a common standard. *Addiction* 2005;100: 299–303. doi: 10.1111/j.1360-0443.2004.00995.x
33. National Institute for Health and Care Excellence. *Training in smoking prevention and cessation.* NICE, 2015.
34. Bauld L, Chesterman J, Ferguson J, Judge K. A comparison of the effectiveness of group-based and pharmacy-led smoking cessation treatment in Glasgow. *Addiction* 2009; 104: 308-16
35. Bauld L, Debbie F. *Local Stop Smoking Services Service and delivery guidance 2014*, 2014 National Centre for Smoking Cessation and Training
36. Sohanpal R, Rivas C, Steed L., MacNeill V, Kuan V, Edwards E, Griffiths C, Eldridge S, Taylor S, Walton R. Understanding recruitment and retention in the NHS community pharmacy stop smoking service: perceptions of smoking cessation advisers. *BMJ Open* 2016;6:e010921. doi:10.1136/bmjopen-2015-010921

For peer review only - http://bmjopen.bmj.com/site/about/guidelines.xhtml

37. Roberts C, Sarangi S. Theme-oriented discourse analysis of medical encounters. *Medical Education* 2005;39,632–640. doi:10.1111/j.1365-2929.2005.02171.x

38. Cavacoa A, Roter D. Pharmaceutical consultations in community pharmacies: utility of the Roter Interaction Analysis System to study pharmacist–patient communication. *IJPP* 2010, 18: 141–148

39. Braun V, Clarke V. *Thematic analysis.* In: Cooper, H., ed. (2012) *APA Handbook of Research Methods in Psychology.* (Vol. 2) Washington, DC: American Psychological Association).

40. Rivas C. *Coding qualitative data*. In, Seale, Clive (ed.) *Researching Society and Culture. Third Edition.* London, GB, Sage

41. Rollnick S, Miller WR. What is Motivational Interviewing? *Behavioural and Cognitive Psychotherapy* 1995;23:325-334

42. Hemingway A. Lifeworld-led care: is it relevant for well-being and the fifth wave of public health action? *Int J Qual Studies on Health and Well-being* 2011;6 (4), Article ID 10364

43. Mischler EG. *The discourse of medicine. The dialectics of medical interview*s, Ablex, Norwood, NJ, 1984

44. Husserl E. *The crisis of European science and transcendental phenomenolog*y, Northwestern University Press, Evanston, Ill, USA, 1970

45. Barry CA, Stevenson FA, Britten N, Barber N, Bradley CP. Giving a voice to the lifeworld. More humane, more effective medical care? A qualitative study of doctor-patient communication in general practice. *Soc Sci Med* 2001; 53, 487–505

46. Edwards D. Extreme case formulations: softeners, investment, and doing nonliteral. *Research on Language and Social Interaction* 2000;33(4), 347–373

47. Pomerantz A. Extreme case formulations: A way of legitimizing claims. *Human Studies* 1986; 9*,* 219–229

48. Shah B, Chewning B. Conceptualizing and measuring pharmacist-patient communication: a review of published studies. *Res Soc Admin Pharm* 2006; 2: 153–185

49. Pilnick A. The interactional organization of pharmacist consultations in a hospital setting: a putative structure. *J Pragmatics* 2001; 33, 1927–1945

50. Pilnick A. “Patient counseling” by pharmacists: advice, information, or instruction? *Sociol Quart* 1999; 40, 613–622

51. Pilnick A. "Patient counselling" by pharmacists: four approaches to the delivery of counselling sequences and their interactional reception. *Soc Sci Med* 2003; 56, 835–849.

52. Garner M, Watson MC. Using linguistic analysis to explore medicine counter assistants' communication during consultations for nonprescription medicines. *Patient Educ Couns* 2007; 65,51–57

53. Stevenson FA. Achieving visibility? Use of non-verbal communication in interactions between patients and pharmacists who do not share a common language. *Sociology of Health & Illness* 2014; 36(5), 1467. DOI:10.1111

54. Stevenson FA, Leontowitsch M, Duggan C. Over-the-counter medicines: professional expertise and consumer discourses. *Sociol Health Illn* 2008;30,913–928

55. Leontowitsch M, Stevenson F, Nazareth I, Duggan C.‘At the moment it is just a couple of eccentrics doing it’: concordance in day-to-day practice. *Int J Pharm Pract* 2005; 13, 273–280

56. Evans SW, John DN. A preliminary investigation of the interactions between UK and US community pharmacists and their prescription clients. *Int J of Pharmacy Practice* 1995; 3: 157–162. doi: 10.1111/j.2042-7174.1995.tb00810.x

57. Pelicano-Romano J, Neves MR, Amado A, Cavaco AM. Do community pharmacists actively engage elderly patients in the dialogue? Results from pharmaceutical care consultations. *Health Expectations* 2015, 18: 1721–1734. doi: 10.1111/hex.12165

For peer review only - http://bmjopen.bmj.com/site/about/guidelines.xhtml

58. Chen J, Britten N. 'Strong medicine': an analysis of pharmacist consultations in primary care. *Fam Pract* 2000;17, 480–483
59. Babalola O, Erhun WO. Pharmacist medication history interview of elderly patients in a teaching hospital in Nigeria. *Int J Pharm Pract* 2001;9, 97–103
60. Salter C. Compliance and concordance during domiciliary medication review involving pharmacists and older people. Sociol Health Illn, 32 (2010), pp. 21–36
61. Hargie OD, Morrow NC, Woodman C. Pharmacists' evaluation of key communication skills in practice. *Patient Educ Couns* 2000;39, 61–70
62. Dyck A, Deschamps M, Taylor J. Pharmacists' discussions of medication side effects: a descriptive study. *Patient Educ Couns* 2005; 56, 21–27
63. Nguyen HT. Boundary and alignment in multiparty conflict talk. *J Pragmatics* 2011; 43, 1755–1771
64. Bissell P, Ward P, Noyce PR. Appropriateness measurement: application to advice-giving in community pharmacies. *Soc Sci Med* 2000;51, 343–359
65. Skoglund P, Isacson D, Kjellgren DI. Analgesic medication–communication at pharmacies. *Patient Educ Couns* 2003; 51,155–161
66. Montgomery AT, Kettis Lindblad A, Eddby P, Soderlund E, Tully MP, Kalvemark Sporrong S. Counselling behaviour and content in a pharmaceutical care service in Swedish community pharmacies. *Pharm World Sci* 2010;32,455–463
67. Watermeyer J,Penn C. "Tell me so I know you understand": pharmacists' verification of patients' comprehension of antiretroviral dosage instructions in a cross-cultural context. *Patient Educ Couns* 2009;75, 205–213
68. Watermeyer J. "Now here come the pills that are going to save your life": pharmacists' discussions of antiretroviral drugs in a context of life and death. *AIDS Care* 2011; 23,807–813
69. Watermeyer J, Penn C. The organization of pharmacist-patient interactions in an HIV/aids clinic. *J Pragmatics* 2009, 41,2053–2071
70. Ylanne V, John DN. Roles of medicines counter assistants in advice giving in community pharmacies: a discourse analysis. *Pharm World Sci* 2008;30, 199–2077
71. Paluck EC, Green LW, Frankish CJ, Fielding DW, Haverkamp B. Assessment of communication barriers in community pharmacies. *Eval Health Prof* 2003 26: 380
72. Habermas J. *The theory of communicative action, Reason and the rationalization of society,* vol. 1. London: Heinemann, 1984
73. Seale C, Rivas C, Kelly M. The challenge of communication in interpreted consultations in diabetes care: a mixed methods study. *The British Journal of General Practice* 2013;63(607):e125-e133. doi:10.3399/bjgp13X663082.
74. Webb H. 'I've put weight on cos I've bin inactive, cos I've 'ad me knee done': moral work in the obesity clinic. *Sociology of Health and Illness* 2009; 31 (6), 854–871
75. Heritage J, Lindstrom A. Motherhood, medicine, and morality: scenes from a medical encounter. *Research on Language and Social Interaction* 1998; 31, 397–438
76. Heritage J, Robinson JD. *Accounting for the visit: giving reasons for seeking medical care*. In: J. Heritage, D.W. Maynard (Eds.), Communication in medical care: Interaction between primary care physicians and patients, Cambridge University Press, Cambridge, 2006, pp. 48–85
77. McMullen LM, Herman J. Women's accounts of their decision to quit taking antidepressants. *Qualitative Health Research* 2009;19 (11), 1569–15
78. Peräkylä A. Authority and accountability: the delivery of diagnosis in primary health care. *Social Psychology Quarterly* 1998;61 (4), 301–320
79. Roberts C, Sarangi S, Moss B. Presentation of self and symptoms in primary care consultations involving patients from non-English speaking backgrounds. *Communication & Medicine* 2004; 1 (2),159–169

For peer review only - http://bmjopen.bmj.com/site/about/guidelines.xhtml

80. Stephens C, Breheny M. Menopause and the virtuous woman: the importance of the moral order in accounting for medical decision making. *Health* 2008; 12, 7–24
81. Seale C, Rivas C, Al-Sarraj H, Webb S, Kelly M. Moral mediation in interpreted health care consultations, *Social Science & Medicine* 2013; 98, 141-148, ISSN 0277-9536, http://dx.doi.org/10.1016/j.socscimed.2013.09.014.
82. Britten N, Stevenson FA, Barry CA, Barber N, Bradley CP. Misunderstandings in prescribing decisions in general practice: qualitative study *BMJ* 2000; 320 :484.
83. Trinkaus Zagzebski L. Epistemic authority: a theory of trust, authority, and autonomy in belief, Oxford University Press, Oxford, 2012
84. Goffman E. *Interaction Ritual: Essays on face-to-face behavior.* (New York: Doubleday Anchor, 1967) (London: Allen Lane, 1972)
85. Lancaster T, Stead LF. Individual behavioural counselling for smoking cessation. *Cochrane Database of Systematic Reviews* 2005, Issue 2. Art. No.: CD001292. DOI: 10.1002/14651858.CD001292.pub2.
86. West R, Walia A, Hyder N, et al. Behavior change techniques used by the English Stop Smoking Services and their associations with short-term quit outcomes. *Nicotine Tob Res* 2010;12(7):742-7. doi: 10.1093/ntr/ntq074.

Figure 1: Analysis process.

Figure 2: Tentative model of misalignment in the community pharmacy NHS Stop Smoking service consultation.

Funding: NIHR Programme Grants for Applied Research Programme (RP-PG-0609-10181). Disclaimer This article presents independent research funded by the National Institute for Health Research (NIHR) under its Programme Grants of Applied Research Programme. The views expressed are those of the authors and not necessarily those of the NHS, the NIHR or the Department of Health.

Data sharing statement. This study analyses qualitative data and the advisers and smokers did not consent to have their full consultation transcripts made publicly available. They consented for their data to be stored for 5 years, as part of the 5-year programme grant project, and to be used in this or other studies directly linked to the 5-year project. Supporting excerpts from the raw data (quotes from consultations) are available within the text of the paper. Full transcripts (with identifying information removed) will be available, following completion of the project, on request from the study guarantor, Robert Walton, r.walton@qmul.ac.uk

For peer review only

For peer review only - http://bmjopen.bmj.com/site/about/guidelines.xhtml

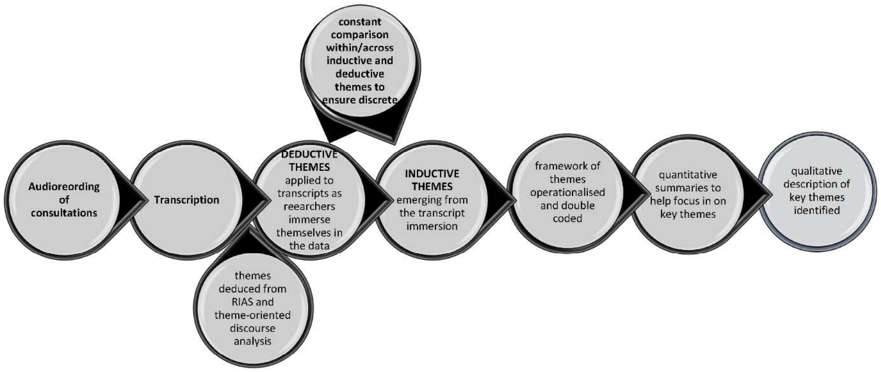

Figure 1: Analysis process

297x210mm (300 x 300 DPI)

For peer review only - http://bmjopen.bmj.com/site/about/guidelines.xhtml

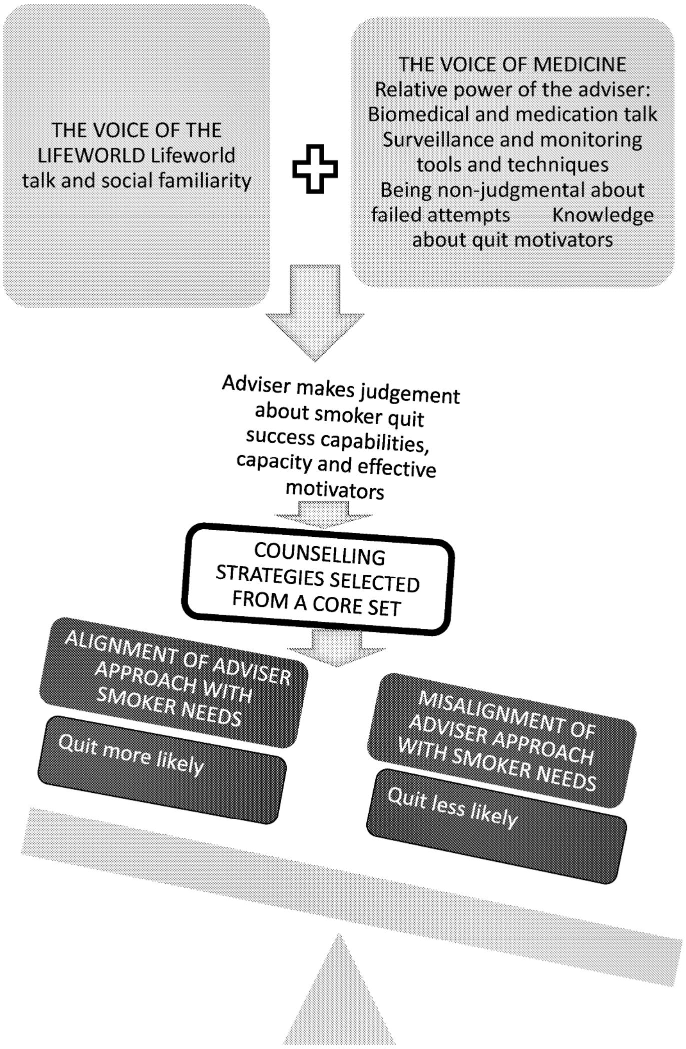

Figure 2: Tentative model of misalignment in the community pharmacy NHS Stop Smoking service consultation.

296x419mm (300 x 300 DPI)

COREQ (COnsolidated criteria for REporting Qualitative research) Checklist

A checklist of items that should be included in reports of qualitative research. You must report the page number in your manuscript where you consider each of the items listed in this checklist. If you have not included this information, either revise your manuscript accordingly before submitting or note N/A.

Topic	Item No.	Guide Questions/Description	Reported on Page No.
**Domain 1: Research team and reflexivity**			
Personal characteristics			
Interviewer/facilitator	1	Which author/s conducted the interview or focus group?	N/A
Credentials	2	What were the researcher's credentials? E.g. PhD, MD	
Occupation	3	What was their occupation at the time of the study?	
Gender	4	Was the researcher male or female?	
Experience and training	5	What experience or training did the researcher have?	
Relationship with participants			
Relationship established	6	Was a relationship established prior to study commencement?	
Participant knowledge of the interviewer	7	What did the participants know about the researcher? e.g. personal goals, reasons for doing the research	
Interviewer characteristics	8	What characteristics were reported about the inter viewer/facilitator? e.g. Bias, assumptions, reasons and interests in the research topic	
**Domain 2: Study design**			
Theoretical framework			
Methodological orientation and Theory	9	What methodological orientation was stated to underpin the study? e.g. grounded theory, discourse analysis, ethnography, phenomenology, content analysis	5
Participant selection			
Sampling	10	How were participants selected? e.g. purposive, convenience, consecutive, snowball	4
Method of approach	11	How were participants approached? e.g. face-to-face, telephone, mail, email	4
Sample size	12	How many participants were in the study?	6
Non-participation	13	How many people refused to participate or dropped out? Reasons?	6
Setting			
Setting of data collection	14	Where was the data collected? e.g. home, clinic, workplace	4
Presence of non-participants	15	Was anyone else present besides the participants and researchers?	4
Description of sample	16	What are the important characteristics of the sample? e.g. demographic data, date	7
Data collection			
Interview guide	17	Were questions, prompts, guides provided by the authors? Was it pilot tested?	N/A
Repeat interviews	18	Were repeat inter views carried out? If yes, how many?	
Audio/visual recording	19	Did the research use audio or visual recording to collect the data?	
Field notes	20	Were field notes made during and/or after the inter view or focus group?	
Duration	21	What was the duration of the inter views or focus group?	
Data saturation	22	Was data saturation discussed?	
Transcripts returned	23	Were transcripts returned to participants for comment and/or	

For peer review only

Topic	Item No.	Guide Questions/Description	Reported on Page No.
		correction?	
**Domain 3: analysis and findings**			
Data analysis			
Number of data coders	24	How many data coders coded the data?	5
Description of the coding tree	25	Did authors provide a description of the coding tree?	9
Derivation of themes	26	Were themes identified in advance or derived from the data?	5
Software	27	What software, if applicable, was used to manage the data?	n/a
Participant checking	28	Did participants provide feedback on the findings?	n/a
Reporting			
Quotations presented	29	Were participant quotations presented to illustrate the themes/findings? Was each quotation identified? e.g. participant number	12
Data and findings consistent	30	Was there consistency between the data presented and the findings?	12
Clarity of major themes	31	Were major themes clearly presented in the findings?	12
Clarity of minor themes	32	Is there a description of diverse cases or discussion of minor themes?	12

Developed from: Tong A, Sainsbury P, Craig J. Consolidated criteria for reporting qualitative research (COREQ): a 32-item checklist for interviews and focus groups. *International Journal for Quality in Health Care*. 2007. Volume 19, Number 6: pp. 349 – 357

Once you have completed this checklist, please save a copy and upload it as part of your submission. DO NOT include this checklist as part of the main manuscript document. It must be uploaded as a separate file.

For peer review only
